# SYMPOSIUM PRESENTATIONS

**DOI:** 10.3402/mehd.v23i0.17462

**Published:** 2012-05-23

Mongolian gerbils are frequently used to study *Helicobacter pylori*-induced gastritis and its consequences. The presence of some gastric microbiota with a suppressive effect on *H. pylori* suggests inhibitory gastric bacteria against *H. pylori* infection. The aim of the present study was to analyze the microbiota in the stomach of Mongolian gerbils with *H. pylori* infection.

In the first infection experiment, according to the frequency of detection of *H. pylori* urea in fecal samples, the infected gerbils were divided into three groups (frequently detected, moderately detected and infrequently detected). *Eubacterium limosum* and *Lactobacillus* spp. were isolated from the frequently detected group and infrequently detected group, respectively. In the second infection experiment, the gastric mucosa samples of *H. pylori* negative and positive gerbils were orally inoculated to five (group 1) and six (group 2) gerbils, respectively, and these gerbils were challenged with *H. pylori* infection. Colonization rate (40%) of *H. pylori* in group 1 was lower than that (67%) in group 2 gerbils. Culture filtrate of gastric mucosa samples of group 1 gerbils inhibited the in vitro growth of *H. pylori*. Three lactobacilli species of *Lactobacillus reuteri*, *Lactobacillus johnsonii* and *Lactobacillus murinus* were isolated by anerobic culture from the gerbils in groups 1 and 2 and identified by genomic sequencing method. Although these lactobacilli showed no inhibitory effect on adhesion of *H. pylori* to gastric cells, it was demonstrated that *L. murinus* exhibited an inhibitory effect on the in vitro growth of *H. pylori*.

Microbial ecology between *H. pylori* and gastric microbiota in Mongolian gerbil was analyzed by two infection experiments. The results from the experiments, the presence of gastric bacteria with inhibitory effect on *H. pylori*, were detected. It was suggested that *L. murinus* isolated from gastric mucosa with inhibitory effect on *H. pylori* might be a novel probiotics candidate against *H. pylori* infection. Recent research data obtained by molecular analysis of gastric microbiota will be also presented in the lecture.
