# Supplementary material for: SYMPOSIUM PRESENTATIONS
Source: Microb Ecol Health Dis. 2012 May 23;23:10.3402/mehd.v23i0.17462. doi: 10.3402/mehd.v23i0.17462 (PMC3747742; doi:10.3402/mehd.v23i0.17462)
Supplement: Related texts

# Sub article: Hematopoietic stem cell transplantation (HSCT)

**Published:** 2012-05-23

**Authors:** Shunichi Kato

**Affiliations:** Department of Cell Transplantation, Tokai University School of Medicine, Isehara, Japan

Bone marrow transplantation (BMT) was started in 1970s in Japan, and Seattle-type regimen was introduced by several BMT centers in early 1980s.

Peripheral blood stem cell transplantation (PBSCT) and cord blood transplantation (CBT) were added into new HSCT in 1990s.

Unrelated HSCT became available through the Japan Marrow Donor Program (JMDP) established in 1991 and the Japan Cord Blood Bank Network (JCBBN) established in 1999.

The numbers of allogeneic HSCT have been increased year by year and reached 4500 per year in 2008, while the numbers of autologous HSCT have been almost stable in the last 10 year, approximately 1500 per year (Fig. 1).

**Fig. 1:** Transplant activity in Japan, 1980–2008.

The source of stem cells in related HSCT is balanced by BM and PBSC in the last 10 years in adults while BM is the major source in children (Fig. 2).

**Fig. 2:** Transplant activity in Japan related allogeneic 1980–2008

The numbers of unrelated HSCT have been increasing steadily. The cumulative transplant numbers exceed 12,500 in UBMT and 7000 in UCBT. In 2011, UBMT was 1200 and UCBT was 1000 (Fig. 3). UPBSCT was first introduced in 2011, and only a few transplants were performed until now.

**Fig. 3:** UBMT and UCBT in Japan 1993–2010.

Japan is isolated from the rest of the world, and the population is genetically homogeneous. There has been no major blood mixture in the last 1000 years. Therefore, the chance to find an HLA-matched BM or CB donor is much higher than in other ethnic groups.

UCBT has been applied to many adult patients, and the number of UCBT in Japan is one-third of that in the whole world. There are many excellent reports on UCBT from Japan, some of which will be presented and discussed in details in the lecture.

# Sub article: The current situation and progress in haploidentical hematopoietic stem cell transplantation

**Published:** 2012-05-23

**Authors:** Xiaojun Huang

**Affiliations:** 1 Peking University Institute of Hematology, Beijing, China; 2 Department of Hematology, Peking University People's Hospital, Beijing, China

Extensive ex vivo T-cell depleted (TCD) or unmanipulated haploidentical transplantation provides benefits of rapid and near universal donor availability for patients without a HLA-identical sibling donor or those who urgently need transplant. However, CD34 selected haplotype mismatched transplantation was limited by delayed immune reconstitution (IR), although this protocol has now been an acceptable approach. Recently, Peking University researchers developed a novel approach to HLA-mismatched/haploidentical blood and marrow transplantation without in vitro TCD (GIAC protocol). Our clinical data showed that G-BM combined with PBSC from haploidentical family donors, without in vitro TCD, might be a good source of stem cells for allo-HSCT. Applying this transplant setting can achieve comparable outcomes with HLA-identical sibling transplantation and even better graft-versus-leukemia effect. To improve the outcomes of patients, we modified the donor lymphocyte infusion (DLI) protocol by using G-CSF-mobilized PB progenitor cells (GPBPCs) instead of traditional steady-donor lymphocytes in therapeutic infusion and further demonstrated the feasibility of applying this strategy against leukemia recurrence from therapeutic DLI to prophylaxis DLI for patients with advanced hematological malignancies undergoing haploidentical transplants. Moreover, much progress has also been made in controlling graft-versus-host disease (GVHD) through manipulating the cell contents or function of graft using various kinds of stimulating factors and improving the recovery of IR via novel approach.

# Sub article: Emerging roles of noninherited maternal alloantigens (NIMAs) and inherited paternal alloantigens (IPAs) in HLA-mismatched hematopoietic cell transplantation

**Published:** 2012-05-23

**Authors:** Tatsuo Ichinohe

**Affiliations:** Division of Hematology, Respiratory Medicine and Oncology, Department of Internal Medicine, Faculty of Medicine, Saga University, Saga, Japan

HLA compatibility between the donor and recipient has long been recognized as an essential prerequisite for successful allogeneic hematopoietic cell transplantation (HCT). However, increasing needs for allogeneic HCT have now expanded the availability of alternative stem cell sources such as unrelated cord blood units and HLA-haploidentical–related family members.

During pregnancy, fetal immune system needs to suppress harmful responses against noninherited maternal alloantigens (NIMAs), and vice versa, maternal immune system must tolerate inherited paternal alloantigens (IPAs) of the fetus, suggesting the presence of natural mechanisms to generate a form of bidirectional immune tolerance between the mother and her HLA-haploidentical fetus. Nevertheless, a substantial proportion of mothers are believed to subsequently become sensitized against IPAs and umbilical cord blood of their offspring is shown to paradoxically contain cytotoxic T-cells against NIMAs. Therefore, we hypothesized that better understanding of the fetomaternal immunology may shed new light on alternative strategies for HLA-mismatched allogeneic HCT.

In 1950s, (1) first provided experimental evidence that the introduction of maternal antigens into the fetus during pregnancy gives rise to a form of immunologic hyporesponsiveness to maternal alloantigens later in life. To elucidate the mechanism by which immune tolerance against maternal alloantigens is maintained in adults, we developed a murine model of NIMA-mismatched HCT by use of the F1 xP backcross breeding model. Intriguingly, CD4^+^ T-cells from NIMA-exposed offspring compared with those from NIMA-nonexposed controls showed reduced proliferative responses and IFN-γ-production in response to NIMA-expressing allogeneic antigen presenting cells. Furthermore, allogeneic HCT from a NIMA-exposed mouse to MHC-incompatible but NIMA-expressing recipients was associated with compromised severity of graft-versus-host disease and superior survival in a NIMA-specific manner. Notably, such tolerogenic effect was abolished when the hematopoietic cell graft from a NIMA-exposed donor was depleted of CD4^+^CD25^+^ T-cells, suggesting that NIMA-specific tolerance is maintained by a subset of T-cells harboring regulatory properties.

We next examined the effect of maternal exposure to IPAs when mothers are used as donors for HCT by use of a similar murine model. CD4^+^ T-cells isolated from IPA-exposed mice compared with those from non-IPA-exposed mice showed comparable proliferative responses to IPA-expressing antigen presenting cells, although maternal cells are generally believed to be sensitized against IPAs. Transplants from IPA-exposed mice to MHC-incompatible IPA-expressing recipients also showed similar but not inferior survival rates compared with transplants from non-IPA-exposed controls

In line with these observations, recent clinical evidence has indicated that the use of NIMA-mismatched or maternal stem cell sources may improve outcomes of allogeneic HCT in selected series of patients. To confirm the presence of such beneficial ‘fetomaternal effects’, a prospective study is warranted to compare the outcomes of transplants using hematopoietic stem cell grafts mismatched for NIMAs/IPAs and those using grafts not mismatched for NIMAs/IPAs.

## References

[1] Billingaham RE, Brent L, Medawar PB (1953). Actively acquired tolerance of foreign cells. Nature.

# Sub article: Properties of sialic acid-binding adhesin of *Streptococcus gordonii*, an oral bacterium as a member of dental plaque organisms and etiological agent for infective endocarditis

**Published:** 2012-05-23

**Authors:** Kiyoshi Konishi, Yukihiro Takahashi

**Affiliations:** Department of Microbiology, Nippon Dental University School of Life Dentistry, Tokyo, Japan

It is currently well known that the poor oral health contribute to many systemic diseases, such as infective endocarditis explained as an odontogenic (dental) focal infection. Viridans streptococci, *Staphylococcus aureus*, enterococci, *Candida albicans*, and others colonize damaged heart valves and frequently identified bacteria acting as etiological agents of infective endocarditis. One of these etiological agents, *Streptococcus gordonii*, is a member of the biofilm community that comprise a numerically prominent group of oral bacteria, which occur primarily on the human tooth surface, commonly referred to as dental plaque. For the mechanism of infective endocarditis, attachment of blood-borne bacteria, which intrude from oral bacterial flora or dental plaque to platelets of target site, is a postulated central event after platelets and fibrin bound to endothelial cells at the site of injury cardiac valves. Some papers indicated erythrocytes also contribute somewhat to the infective endocarditis.

*S*. *gordonii* adhere to saliva-coated hydroxyapatite, an experimental model of the tooth surface, and attach to host cells such as erythrocytes and platelets. A common mechanism in these interactions is to recognize surface-associated host sialoglycoconjugates. Recently, such interactions have been found to involve the binding of streptococcal adhesins identified as large serine-rich glycoproteins to membrane-sialoglycoproteins of host cells. We previously reported that the *S*. *gordonii* DL1 *hsa* gene encoded a large serine-rich repeat protein (Hsa) composed of 2178 amino acid residues. Hsa binds a2-3-linked sialic acid termini of *O*-glycosylated musin-type glycoproteins and consists of an *N*-terminal nonrepetitive region (NR1, containing signal sequence), a serine-rich repeat region (SR1), a second nonrepetitive region (NR2), a second serine-rich repeat region (SR2) and a *C*-terminal cell wall anchoring domain. We also reported that an insertional mutation in *hsa* gene resulted in a significant reduction of the infection rate of the organism and inflammatory reaction in the rat aortic valve with experimental endocarditis, suggesting that the Hsa contributes to the infectivity of the organism for heart valves. We have identified that the receptors of erythrocyte for Hsa is glycophorin A and band 3, using expressed recombinant NR2, a putative binding domain of Hsa, fused with GST in *Escherichia coli* BL21. We have also identified GPIba and GPIIb as platelet receptors for *S*. *gordonii* DL1 Hsa. More recently, we have reported that monocyte stimulated with *S*. *gordonii* DL1 rapidly undergo monocyte-to-dendritic cell differentiation through interaction with the Hsa and suggested that this response may be attribute to the initial step in infective endocarditis by oral streptococci.

# Sub article: Intestinal bacterial microbiota: lessons learned

**Published:** 2012-05-23

**Authors:** Marika Mikelsaar

**Affiliations:** Department of Microbiology, University of Tartu, Estonia, Ravila, Tartu, Estonia

In the 1990s, our understanding of microbial diversity in intestinal microbiota expanded to include new taxonomic and functional characteristics. Previously, by cultivation on selective and nonselective media, anaerobes such as *Bacteroides*, eubacteria, bifidobacteria, peptostreptococci and fusobacteria were thought to predominate over the coliforms, *Lactobacillus*/*Enterococcus* and staphylococci (1). However, newly developed molecular technologies have revealed nearly 100-fold greater total numbers of bacteria in the large intestines, with an abundance of 3–12% for some inculturable bacteria, such as *Atopobium*/*Eubacterium, Clostridium coccoides* and the *Clostridium leptum* groups (2). Postgenomic approaches have enabled study of the diversity and functionality of this complex ecosystem using new methods, such as metabolomics, proteomics, transcriptomics and genomics (3). Several environmental factors, such as diet and medical/pharmaceutical interventions, were shown to influence microbiota composition, which is intimately associated with GI function in health and disease. The overall aim has mainly been to identify the intervention(s) responsible for these effects on bacteria on an individual and temporal scale.

Another possibility is to elucidate the impact of some established components of this microbiota on human health indices. Based on the diverse functions of GI microbiota, incrementally obtained knowledge on the expression of microflora-associated characteristics (MAC) (4) in macroorganisms has been further elaborated by studies on microbial-host crosstalk (5). Currently, this crosstalk involves the recognition of self and nonself by toll-like receptors on host dendritic cells and macrophages (6), which shape the maturation of GI cells and the development of innate and obtained immunity. Still, the most important recent discoveries are the elucidation of the mechanisms by which intestinal microbiota impact host metabolism. Several experimental and clinical studies have revealed that intestinal microbiota, which live in mutual, beneficial symbiosis with the host organism, are important regulators of energy uptake and storage (7, 8), suggesting a link between microbiota and metabolic diseases, such as type 2 diabetes and metabolic syndrome.

In our laboratory, we have developed an approach to characterize the health status of different age groups according to different anthropometrical and clinical characteristics, as well as cellular, biochemical and immunological blood and urine indices (8–11). Using this approach, we have obtained statistical normal reference values for nearly 50 health status indices in different age groups in a geographically distinct Estonian population. Furthermore, we have correlated these data with bacteriologically and molecularly assessed *Lactobacillus* sp. compositions and describe some species and strains as risk modulators for increased glucose and triglyceride content in the blood, impaired cholesterol metabolism, high blood pressure, obesity and antimicrobial and immunological competence. The metabolites profile of the defined *Lactobacillus* sp. contains antimicrobial and antioxidative compounds (Mn-superoxide dismutase, glutathione system), as well as nitric mono-oxide and polyamines (12). These findings have aided the prediction and manipulation of the functionality of microbiota, resulting in the ability to transform host ecosystems using pinpointed biotechnological applications of probiotics.

## References

[1] McFarland LV (2000). Normal flora:diversity and functions. MEHD.

[2] Zoetendahl EG, Vaughan EE, de Vos W (2006). A microbial world within us. Mol Microbiol.

[3] Zoetendahl EG, Rajili M, de Vos WM (2008). High-throughput diversity and functionality analysisi of the gastrointestinal tract microbiota. Gut.

[4] Midtvedt T, Björneklett A, Carlstedt-Duke B, Gustafsson BE, Hoverstadt T, Lingaas E (1985). The influence of antibiotics upon microflora-associated characteristics in man and mammals. Progr Clin Biol Res.

[5] Falk PG, Hooper LV, Midtvedt T, Gordon JJ (1998). Creating and maintaining the gastrointestinal ecosystems:what we knw and need to know from gnotobiology. Microbiol Mol Biol Rev.

[6] Takeda K, Kaisho T, Akira S (2003). Toll-like receptors. Annu Rev Immunol.

[7] Bäckhed F, Ding H, Wang T, Hooper LV, Koh GY, Nagy A (2004). The gut microbiota as an environmental factor that regulates fat storage. Proc Natl Acad Sci USA.

[8] Turnbaugh PJ, Ley RF, Mahowald MA, Magrini V, Mardis FR, Gordon JJ (2006). An obesity-associated gut microbiome with increased capacity for energy harvest. Nature.

[9] Mikelsaar M, Annuk H, Stsepetova J, Mandar R, Sepp E, Björksten B (2002). Intestinal *Lactobacilli* of Estonian and Swedish children. Microb Ecol Health Dis.

[10] Mikelsaar M, Stsepetova J, Hütt P, Kolk H, Sepp E, Lõivukene K (2010). Intestinal Lactobacillus sp. is associated with some cellular and metabolic characteristics of blood in elderly people. Anaerobe.

[11] Stsepetova J, Sepp E, Kolk H, Lõivukene K, Songisepp E, Mikelsaar M (2011). Diversity and metabolic impact of intestinal Lactobacillus sp. in healthy adults and the elderly. Br J Nutr.

[12] Kullisaar T, Songisepp E, Aunapuu M, Kilk K, Arend M, Mikelsaar M, Rehema A, M Zilmer M (2010). Complete glutathione system in probiotic *Lactobacillus fermentum* ME-3. Appl Biochem Microbiol.

# Sub article: Molecular ecology of butyrate-producing bacteria from the human gut

**Published:** 2012-05-23

**Authors:** Petra Louis

**Affiliations:** Rowett Institute of Nutrition and Health, University of Aberdeen, Bucksburn, Aberdeen, United Kingdom

The human large intestine is colonized by a highly diverse microbial community, which is dominated by bacteria belonging to Gram-negative Bacteroidetes and Gram-positive Firmicutes. The gut microbiota receives most of its energy from dietary carbohydrates that cannot be digested by the human host and reach the colon. The quantity and type of dietary carbohydrate ingested is likely to influence the composition and activity of the gut microbiota, with potential consequences for human health. Carbohydrate fermentation by the microbial community as a whole leads to the accumulation of three main short-chain fatty acids: acetate, propionate and butyrate. Other fermentation products such as formate, lactate, succinate and branched-chain fatty acids may accumulate to a lesser degree.

Butyrate has received special attention due to its role as main fuel for the colonic wall and its anticarcinogenic and anti-inflammatory effects. Butyrate producers belong to several different clostridial clusters within Firmicutes. Two major groups of butyrate producers are bacteria related to *Faecalibacterium prausnitzii* within clostridial cluster IV and *Eubacterium rectale* and *Roseburia* spp. within clostridial cluster XIVa. Another cluster XIVa group that is of functional importance for the conversion of lactate to butyrate is lactate-utilizing butyrate producers related to *Eubacterium hallii* and *Anaerostipes* spp. Further species of butyrate producers are phylogenetically interspersed with nonbutyrate-producing bacteria, which make it difficult to monitor the butyrate-producing capacity of the gut microbiota using molecular approaches based on the 16S rRNA gene. We, therefore, targeted a functional gene involved in butyrate metabolism in the majority of human butyrate producers, butyryl-CoA:acetate CoA-transferase, to investigate the diversity of this functional group and monitor changes in response to prebiotic supplementation in healthy human volunteers (1). Thirty-two different operational taxonomic units (OTUs, <98% sequence identity at DNA level) were detected in fecal samples from 10 volunteers, with each volunteer carrying between 6 and 17 different OTUs. The most prevalent OTUs belonged to the *E. rectale*/*Roseburia* spp. group, *E. hallii* and as-yet unnamed strain SS2/1, all within clostridial cluster XIVa. The majority of sequences (88%) belonged to 12 OTUs that were closely related to cultured isolates, while the remaining 12% of sequences belonged to 20 OTUs without cultured representatives. Thus, OTUs with cultured representatives were mostly abundant, while less-abundant OTUs mostly did not match cultured isolates. This indicates that the lack of cultured isolates for many gut bacteria identified by molecular approaches may be due to their low abundance rather than an inherent unculturability. Supplementation with the prebiotic inulin led to a significant increase in sequences related to *F. prausnitzii*, which confirmed previous microbiota analysis based on the 16S rRNA gene (2). Within the *E. rectale*/*Roseburia* group, a shift in species composition was noted upon inulin consumption with a significant decrease in *Roseburia inulinivorans* and *Roseburia hominis* and a trend toward higher levels of *E. rectale*. Furthermore, sequences related to strain SS2/1 also showed a trend toward an increase after inulin consumption, which could be confirmed by 16S rRNA gene-based qPCR analysis. These results indicate that the effect of prebiotics on the gut microbiota is more complex than originally thought.

The butyryl-CoA:acetate CoA-transferase gene sequence targeted here is most closely related to 4-hydroxy-butyrate CoA-transferases from several *Clostridium* species. We were unable to detect this gene in the human gut butyrate producer *Eubacterium cylindroides*, which belongs to clostridial cluster XVI. Phosphotransbutyrylase and butyrate kinase, which are used by some gut bacteria to generate butyrate instead of butyryl-CoA:acetate CoA-transferase, could also not be detected. However, we have recently identified another CoA-transferase gene more closely related to propionate CoA-transferases in clostridial cluster XVI isolates from the human and the chicken gut (3). It appears therefore that different types of CoA-transferase genes may have evolved in different bacterial lineages to perform the last step of butyrate generation.

## References

[1] Louis P, Young P, Holtrop G, Flint HJ (2010). Diversity of human colonic butyrate-producing bacteria revealed by analysis of the butyryl-CoA:acetate CoA-transferase gene. Env Microbiol.

[2] Ramirez-Farias C, Slezak K, Fuller Z, Duncan A, Holtrop G, Louis P (2009). Effect of inulin on the human gut microbiota: stimulation of *Bifidobacterium adolescentis* and *Faecalibacterium prausnitzii*. Br J Nutr.

[3] Eeckhaut V, Van Immerseel F, Croubels S, De Baere S, Haesebrouck F, Ducatelle R (2011). Butyrate production in phylogenetically diverse *Firmicutes* isolated from the chicken cecum. Microbial Biotechnol.

# Sub article: Study of human hematopoietic stem cell aging using immune-deficient mice

**Published:** 2012-05-23

**Authors:** Kiyoshi Ando

**Affiliations:** Department of Hematology and Oncology, Tokai University School of Medicine, Isehara, Japan

Stem cells of highly regenerative organs including blood are susceptible to endogenous DNA damage caused by both intrinsic and extrinsic stress. Response mechanisms to such stress equipped in hematopoietic stem cells (HSCs) are crucial to sustain hematopoietic homeostasis but remain largely unknown. We demonstrate that replication stress induces intracellular elevation of reactive oxygen species (ROS) that results in accumulated and persistent DNA damage in human HSCs both in vitro and in vivo. This accumulation of DNA damage is demonstrated in HSCs of clinical transplant patients and elderly individuals in addition to a xenotransplantation model. The oxidative DNA damage causes premature senescence among HSCs, leading to loss of stem cell function. Importantly, treatment with an antioxidant can antagonize oxidative DNA damage and consequent HSC dysfunction. Our results reveal that ROS play a causative role for DNA damage, and mechanisms of ROS regulation have a major influence on human HSC aging.

# Sub article: Human-induced pluripotent stem cell-derived blood cells toward clinical application

**Published:** 2012-05-23

**Authors:** Naoya Takayama

**Affiliations:** Clinical Application Department, Center for iPS Cell Research and Application, Kyoto University, Kyoto, Japan

The achievement of ‘regenerative medicine’ needs the global and sophisticated system for translation from the basic science to clinical application. We aim to develop the novel blood transfusion and gene- and cellular-therapy, for example, using human-induced pluripotent stem (iPS) cells. We have so far developed the static culture system whereby human iPS cells can be differentiated into the Sac-like structures that concentrate CD34^+^ hematopoietic progenitors, further generating platelets, erythrocytes or T lymphocytes in vitro. In addition, our research program will focus on the developmental strategies for achievement of safe and stable blood supply for transfusion independently of blood donation using immortalized cells derived from human iPS cells. In this symposium, we would like to introduce recent results on platelet generation from human iPS cells and next step toward clinical application.

# Sub article: CMV reactivation and immune reconstitution after CBT

**Published:** 2012-05-23

**Authors:** Satoshi Takahashi

**Affiliations:** Institute of Medical Science, University of Tokyo, Tokyo, Japan

Cytomegalovirus (CMV) reactivation is thought as one of most important problems after cord blood transplantation (CBT). On the other hand, we have achieved good clinical results for adults after CBT with histocompatibility antigen [human leukocyte antigen (HLA)]-mismatched single graft. The one of crucial questions in CBT is whether naivety of lymphocytes could gain antigen-specific cellular immunity during early phase of HLA-mismatched transplant. To answer this, we have analyzed the CMV-specific immune reconstitution process for first 6 months.

Forty adults has received myeloablative regimens including 12 Gy of total body irradiation followed by CBT and a standard cyclosporine and methotrexate combination as GVHD prophylaxis in the Institute of Medical Science, University of Tokyo (IMSUT) and in nine different facilities, which participated for the prospective study using IMSUT regimen. CMV-specific CD4^+^ and CD8^+^ T cell recoveries were assessed by detection of interferon-gamma (IFN-g) producing cells with CMV antigen stimulation using intracellular cytokine staining or tetramers for CMV pp65 in whom HLA-A0201, -A0206 or -A2402 positive patients. The positive was defined as >0.03% IFN-g positive cells among CD4^+^ or CD8^+^ T cell population and >0.01% positive in tetramer assay.

CMV-reactive (IFN-g positive) CD4^+^ T cells were detected in 65% at 1 month, 88% at 2 months, 92% at 3 months, 92% at 4 months and 95% at 6 months after CBT, which were comparable to CMV-positive age-adjusted healthy control (100%). CMV-reactive (IFN-g positive) and CMV-specific (tetramer-positive) CD8^+^ T cells were detected in 53% and 5% at 1 month, 71% and 44% at 2 months, 68% and 36% at 3 months, 75% and 50% at 4 months and 65% and 50% at 6 months (39% and 67% in the control). Next, we looked the effect of HLA disparity (HLA-DR for CD4^+^ and HLA-A/-B for CD8^+^ T cell) in graft-versus-host direction with low resolution typing (LRT) and in high resolution typing (HRT). CMV-reactive CD4^+^ T cells were detected in 94% with matched (0MM), 81% with one antigen mismatched (1AMM) in LRT and 100% with 0MM, 89% with 1AMM, 80% with 2AMM in HRT at 2 months. CMV-specific CD8^+^ T cells were detected in 33% with 0MM, 38% with 1AMM, 56% with 2AMM in LRT and 38% with 1AMM, 50% with 2AMM, 67% with 3AMM in HRT at 2 months, respectively.

Postthymic naive T cells in cord blood might obtain memory and effector function in vivo with antigen-specific manner during early phase of posttransplant independent on effect of HLA compatibility. When we evaluated the impact of positive antigenemia on clinical outcomes of CBT, HLA disparities were not affected to high frequency of positive CMV antigenemia results. Significant longer hospitalization was needed in high-frequent CMV-reactivated patients after transplantation; however, cumulative incidences of neutrophil and platelet recoveries, of GVHD, of relapse and of nonrelapse mortality were not affected by high-frequent CMV positivity of post-CBT.

# Sub article: The immunobiology of hematopoietic stem cell transplantation

**Published:** 2012-05-23

**Authors:** Anders Fasth

**Affiliations:** Department of Pediatrics, University of Gothenburg, Gothenburg, Sweden; Department of Pediatrics, The Queen Silvia Children's Hospital, Göteborg, Sweden

Hematopoietic stem cell transplantation (HSCT) is a unique situation in the sense that a foreign immune system is introduced into the host with the ultimate goal to replace the host's hematopoiesis including its immune system. This to either allow repair of a faulty hematopoietic system, such as in case of congenital immunodeficiencies and hemoglobulinopathies, or the cure of malignancies through a combination of high dose cytostatic treatment (conditioning therapy) and immunologic rejection of the malignant clone.

Two immune systems in one host open for many complications and have consequences for immune reconstitution after the transplantation.

The most serious complications are rejection of the transplanted cells or the rejection of the host itself, a process called graft-versus-host reaction (GvH), by the incoming T cells. As only a limited amount of stem cells together with more mature cells are transplanted, the balance would favor rejection of the transplanted cells if the host's bone marrow is not ablated, immunosuppressed or, as in case of severe combined immunodeficiency, lacks the capacity to mount an immunoreaction. The most important risk factor for GvH is human leukocyte antigens (HLA) disparity between the host and the donor. HLA is an enormously varied set of cell surface molecules with billions of variants, whose genes are all closely clustered in chromosome 6. There is a 25% chance that siblings are HLA identical.

The pathophysiology of GvH is complicated. Some examples: (1) it involves also the immune system of the patient. For example, the inflammatory process elicited by the cytostatic treatment before the HSCT upgrades HLA class II expression on many cell surfaces, making them an optimal target for T cells in the incoming graft. (2) Also, the inflammatory process with production of proinflammatory cytokines activates the adaptive immune system of the graft. (3) Not only T cells are important but also NK cells and their specific set of natural killer immunoglobulin-like inhibitory and activating receptors (KIR). (4) An important target of the GvH is the thymus. The damage to the thymus will prolong the immune reconstitution of the patient and might leave her with a life-long immunodeficiency. (5) Furthermore, factors such as host and donor age, ongoing infections, cell dose, stem cell source and others are important.

Finally, we must accept that GvH is an important part of the graft-versus-leukemia/tumor effect (GvL or GvT). GvH is a double edge sword that on one hand causes destruction and much suffering to the patient but, on the other hand, diminish the risk for relapse of the malignancy. To harness the GvH and separate it from GvL is a goal that is the subject for intense research.

The immune reconstitution post-HSCT takes long time, and as a consequence, infectious complications are common and contribute significantly both to morbidity and mortality. At the time of the transplantation, the patient is severely immunodeficient due to the conditioning regimen and/or the underlying disease and its treatment. The immune reconstitution after HSCT occur through expansion of T cells in the graft and more efficient through the egress of new T cells that have been expanded and educated in the host's thymus. As thymus starts to involve after puberty, the efficacy of thymus education and formation of new naïve T cells will diminish with increasing age and increase the risk for infectious complication in older patients. Experimentally, it has been shown that epithelial growth factor 7 has a protective role. Much research is devoted to protect the thymus from the damage of conditioning and GvH and to increase the output of new T cells from the thymus to enhance and speed up immune reconstitution. Other attempts to treat or prevent infections are the production of specific cytotoxic T cells directed to infectious agents such cytomegalovirus (CMV) and Epstein-Barr virus (EBV).

A swift establishment of the new stem cells also diminishes the risk for relapse of malignancy, even without overt GvH, pointing to a GvL effect separate from GvH. One important factor for early immune reconstitution is the cell dose. A high cell dose is important, and the low stem cell number, together with the naïve status of the T cells, is a major drawback of umbilical cord blood as source of stem cells.

# Sub article: Microbial metabolic functions in Crohn's disease patients

**Published:** 2012-05-23

**Authors:** Elisabeth Norin

**Affiliations:** Department of Microbiology Tumor and Cell Biology (MTC), Karolinska Institutet, Stockholm, Sweden

The American physician Burrill Crohn described in 1932 a clinical and pathological entity in some patients with abdominal GI symptoms, and this disease has later been given the name Crohn's disease (CD) (1). The disease occurs anywhere from the mount to anus, however, most often in the terminal ileum and/or colon, and it is characterized pathologically by transmural inflammation, deep linear ulceration and often granulomas.

In Sweden, about 25,000 patients are diagnosed as CD patients – approximately 400 persons get the diagnose yearly and those are equally distributed within males and females, most of them 20–30 years old, but the disease is also diagnosed in some few children.

There is no clear cause of the etiology of the disease – however, increasing evidence suggests that a combination of host genetics and the composition/function of the gut microbiota are factors at work (2). Several bacteria species have previously been claimed to be involved, but current hypotheses includes the theory that an unbalanced antigenic microbial stimulation could be one reason for the disease development. A dysbalance of the intestinal microflora do influence on, e.g., the host immunological response, thus causing mucosa alterations.

In the 1980s, we and other demonstrated a decreased inactivation of intestinal tryptic activity (3, 4), and we hypothesized, there is a reduced amount or absence of trypsin-degrading microbes. Since then, data indicating that alterations in composition and function of the intestinal microbiota together with impaired epithelial barrier functions are involved in the disease (5, 6).

As it is known that the host genotype partly determines the microbial community composition in man, our aim has been to apply different ways of attach the question of intestinal flora composition and function in four patients with diagnosed CD. Our group at Karolinska Institutet in the ‘2 kg feces party’ has investigated some microflora-associated characteristics (7) in fecal samples from these patients. Other members of our party investigated both biopsies and fecal samples from the same patients using a culture-independent technique based on molecular biology methods (8) and traditional microbiological culturing techniques.

By applying the different techniques, we found a significant difference in the inactivation/degradation of tryptic activity in the CD patient fecal samples. Results from the other groups in our party using other techniques found that the CD patients have lower number of bacteroides in their intestinal and fecal flora. Thus, a lack of functionally active *Bacteroides distasonis* most probably is one factor involved in the disease development, as it previously has been shown that bacterial strains belonging in the Bacteroides from man as well as animals are able to break down trypsin, both in vivo and in vitro (9). We conclude that the altered pattern of fecal tryptic activity indicate either absence of functionally active bacteroides, lack of other trypsin-degrading bacteria or alterations in intestinal production of microbial or pancreatic secretory trypsin inhibitors (PSTI), thereby indicating the possibility that CD might be due to absence of some metabolically active microbes; in contrast to a more general opinion that presence of some specific microbes are involved in the pathogenesis of CD.

In conclusion, our observations of high levels of fecal tryptic activity found in CD patients could indicate a lack of bacterial-driven breakdown of trypsin – however, the small number of patients so far studied does not allow us to draw any strong conclusions of to what extent these alterations play a role in the etiopathogenes of CD. Our findings in the present materials indicate that CD patients have functional alterations in their intestinal microbiome in parallel with an immunological dysfunction.

The 2 kg party is an interdisciplinary network at Karolinska Institutet dedicated to explore involvement of various host–intestinal microbial interactions in human health and disease.

**Fig. 1:** Fecal tryptic activity.

## References

[1] Crohn BB (1967). Granulomatous disease of the small and large bowel. A historical survey. Gastroenterology.

[2] Sartor RB (2006). Mechanisms of disease: pathogenesis of Crohn's disease and ulcerative colitis. Nat Clin Pract Gastroenterol Hepatol.

[3] van der Merve JP, Mol GJJ (1982). Levels of trypsin and a-chymotrypsin in feces from patients with Crohn's disease. Digestion.

[4] Bergstrand LO, Gustafsson BE, Holstrom B, Norkin KE (1981). The phsyiolgical activity of human ileal flora in patients with Crohns disease and ulcerative colitis evaluated by determination of germfree animal characteristics. Acta Chir Scand.

[5] Willing B, Halfvarson J, Dicksved J, Rosenquist M, Järnerot G, Engstrand L (2009). Twin studies reveal specific imbalances in the mucosa-associated microbiota of patients with ileal Crohn's disease. Inflamm Bowel Dis.

[6] Dicksved J, Halfvarson J, Rosenquist M, Järnerot G, Tysk C, Apajalahti J (2008). Molecular analysis of the gut microbiota of identical twins with Crohn's disease. ISME J.

[7] Norin E, Midtvedt T, Ouwehand A, Vaughan EE (2006). Born germfree – microbial dependent.

[8] Zabarovsky E, Petrenko L, Protopopov A, Vorontsova O, Kutsenko AS, Zhao Y (2003). Restriction site tagged microarrays (RST): a novel technique to identify the species composition of complex microbial systems. Nucleic Acids Res.

[9] Ramare F, Hautefort I, Verhe F, Calam J (1995). Influence of inflammatory bowel disease on the distribution and concentration of pancreatic secretory trypsin inhibitor within the colon. Am J Pathol.

# Sub article: Application of metabolomics approaches to study energy metabolism and reveals the hepatic glycogen accumulation in germ-free mice

**Published:** 2012-05-23

**Authors:** Hsiao-Li Chuang, Yen-Te Huang, Chia-Chung Hou, Chi-Chang Huang

**Affiliations:** 1 Germfree & Gnotobiotic Section, Technical Services Division, National Laboratory Animal Center, National Applied Research Laboratories, Taipei 11529, Taiwan, ROC; 2 Sports Nutrition and Biochemistry Laboratory, Graduate Institute of Sports Science, National Taiwan Sport University, Taoyuan, 33301 Taiwan, ROC

Current nutrition research is focusing on health promotion, disease prevention and performance improvement for individuals and communities around the world. The provision of humans with required nutritional ingredients depends on both how well the individual is provided with balanced foods and what state of gut microbiota the host has. Studying the mutually beneficial relationships between gut microbiome and host is drawing ever-increasing attention in biomedical science. Increasing metabolome-based evidences show that gut microbiota can affect host energy balance, especially fat deposition and lipid metabolism, by microbial metabolites such as short-chain fatty acids. Very recently, we used a gas chromatography-mass spectrometry (GC-MS)-based metabolomics approach to reveal the metabolic profile in gut microbiota- lacking mice and suggested that increased gluconeogenesis and glycogenesis lead to glycogen accumulation in the liver (Chuang *et al*., 2011; *J Nutr Biochem*). Our findings also shed light on a new perspective of the role of gut microbiota in energy metabolism and will be useful to help study interactions between gut microbiota and host metabolism.

**Fig. 1:** Metabolomics for gnotobiotic research: from phenotype to pathophysiology.

# Sub article: Microbial ecology and shelf life of ready-to-eat pomegranate arils packaged under modified atmosphere

**Published:** 2012-05-23

**Authors:** Athanasios Alexopoulos, Elisabeth Chatzivassiliou, Stavros Plessas, Maria Alexakoudi, Irene Theodoridou, Maria Fournomiti, Ioanna Mantzourani, Chryssa Voidarou, Elizabeth Stavropoulou, Eugenia Bezirtzoglou

**Affiliations:** 1 Democritus University of Thrace, Faculty of Agricultural Development, Laboratory of Microbiology, Biotechnology and Hygiene, Orestiada, Greece; 2 Agricultural University of Athens, Laboratory of Phytopathology, Athens, Greece; 3 Democritus University of Thrace, Medical School, Alexandroupolis, Greece

The pomegranate (*Punica granatum*) is a fruit tree of great adaptability to adverse climatic conditions; it is able to support severe colds, salinity soils, tolerate droughts and grow in semi-arid zones. These facts, along with the special dietary characteristics of pomegranate, revive its cultivation during the recent years worldwide and also in Greece. Pomegranates are consumed as fresh fruits, as a juice or as syrup in cocktails and pastries (grenadine). Some parts of the fruit are used in tannery due to the increased content in tannins or in the manufacturing process of dyes and coloring pigments. In food industry, the leading product is the minimally processed and modified atmosphere packaged (MAP) ‘ready-to-eat’ arils. Most of the Greek arils production is dedicated for exporting, and thus it is essential to be properly preserved in order to avoid any losses from spoilage and even more to prolong the shelf life and stability leading to competition advantages. Ways of minimal processing and preservation are the individually quick frozen, drying (natural or vacuumed) and modified atmosphere packaging, alone or in combination with sanitizing agents washing, pH modification, use of antioxidants and temperature control (hurdle technology).

In this study, the shelf life and microbiological safety of packaged pomegranate arils of the *Wonderful* variety was evaluated under different atmosphere compositions, with or without prior sanitizing (sodium hypochlorite solution), antioxidants (ascorbic acid) and pH modifier (citric acid).

As our results showed, arils packaged in PET trays without MAP and stored at 5°C were spoiled after a period of 8 to 12 days or when the total microbiological content (cfu/g) reached 6 logs. In contrast, the optimum results were obtained after initial sanitation of the arils (100 ppm sodium hypochlorite solution), pH modification with citric acid and packaging under MAP (15% CO_2_/5% O_2_). Those conditions if combined with an increased quality of the fruit, low postharvest injury incidence and low temperature preservation (∼5°C) could extend the shelf life of the product to 18–20 days.

# Sub article: Germfree animal studies lead to the revelation of new functions of vitamin K

**Published:** 2012-05-23

**Authors:** Michio Komai, Hitoshi Shirakawa

**Affiliations:** Laboratory of Nutrition, Graduate School of Agricultural Science, Tohoku University, Sendai, Japan

Phylloquinone (vitamin K1, VK1) and the menaquinones (MK-n, or vitamin K2, VK2) are naturally occurring forms of vitamin K. Most of the menaquinone analogs are synthesized by microorganisms, but we have reported that MK-4 is unique in being synthesized by the conversion of orally ingested VK1 or menadione (VK3) in the major tissues of germfree rats and mice, which lack their intestinal microflora. According to our previous studies with germfree animals, we could negate Martius’ theory that described the participation of bacterial enzyme of the intestinal flora to this conversion. Last year, another group (1–3) revealed the enzyme responsible for the menadione (VK3) conversion into MK-4, and this is UBIAD1 (Nature, 2010). However, this enzyme cannot catalyze VK1 conversion into MK-4, which means that UBIAD1 is not the actual enzyme for VK1 conversion into MK-4. Thus, we have just restarted efforts to reveal the true enzyme for the naturally occurring VK1 conversion into MK-4. The result of this study will be presented in the near future.

In addition to the in vivo conversion study, MK-4 has been attracting the attention of researchers due to its specific physiological action such as apoptotic activity on osteoclast cells and leukemia cells, etc. We also discovered new functions of MK-4 by using feeding vitamin K-deficient diet model in mice and rats. One outcome of MK-4 is the anti-inflammatory action, and the other is the steroidogenic effect in the testis through the regulation of Cyp11a.

1. MK-4 enhances testosterone production in rats and testis-derived tumor cells.

Vitamin K is essential for the posttranslational modification of various Gla proteins. Although it is widespread in several organs, including the testis, the function of vitamin K in these organs is not well characterized. In this study, we investigated the function of vitamin K in the testis and analyzed its role in steroidogenesis.

Eight-week-old male Wistar rats were fed a diet supplemented with MK-4 (75 mg/kg diet), one of the predominant K2 vitamins present in the testis, for 5 weeks. In vivo testosterone levels of the rats’ plasma and testes were measured by enzyme-linked immunosorbent assay, and in vitro testosterone levels of testis-derived tumor cells (I-10 cells) maintained in Ham's F-10 medium with 10% fetal bovine serum were measured following treatment with MK-4 (0 to 100 mM) at several time points. Testosterone and cellular protein levels were analyzed with respect to their effects on steroidogenesis.

Testosterone levels in the plasma and testes of MK-4-fed rats were significantly increased compared to those of control rats, with no obvious differences in plasma luteinizing hormone levels. Secreted testosterone levels from I-10 cells were elevated by MK-4, but not by vitamin K1, in a dose-dependent manner independent of cAMP treatment. Western blot analysis revealed that expression of CYP11A, the rate-limiting enzyme in steroidogenesis, and phosphorylation levels of protein kinase A (PKA) and the cAMP response element-binding protein were all stimulated by the presence of MK-4. Enhancement of testosterone production was inhibited by H89, a specific inhibitor of PKA, but not by warfarin, an inhibitor of g-glutamylcarboxylation.

2. Vitamin K suppresses the lipopolysaccharide (LPS)-induced expression of inflammatory cytokines in cultured macrophage-like cells.

We previously found that vitamin K suppresses the inflammatory reaction induced by LPS in rats and human macrophage-like THP-1 cells. In this study, we further investigated the mechanism underlying the anti-inflammatory effect of vitamin K by using cultures of LPS-treated human-and mouse-derived cells. All the vitamin K analogs analyzed in our study exhibited varied levels of anti-inflammatory activity. The isoprenyl side chain structures, except geranylgeraniol, of these analogs did not show such activity; warfarin did not interfere with this activity. The results of our study suggest that the 2-methyl-1,4-naphthoquinone ring structure contributes to express the anti-inflammatory activity, which is independent of the Gla formation activity of vitamin K. Furthermore, MK-4, a form of vitamin K2, reduced the activation of nuclear factor kB (NFkB) and inhibited the phosphorylation of IKKa/b after treatment of cells with LPS. These results clearly show that the anti-inflammatory activity of vitamin K is mediated via the inactivation of the NFkB signaling pathway.

## References

[1] Nakagawa K (2010). Nature.

[2] Ohsaki Y (2010). J Nutr Biochem.

[3] Ito A (2011). Lipids Health Dis.

# Sub article: Blood stream infections in allogeneic hematopoietic stem cell transplant recipients: reemergence of Gram-negative rods and increasing antibiotic resistance

**Published:** 2012-05-23

**Authors:** M. Mikulska, V. Del Bono, A.M. Raiola, B. Bruno, F. Gualandi, D. Occhini, C. di Grazia, A. Bacigalupo, Francesco Frassoni, C. Viscoli

**Affiliations:** Centro Cellule Staminali e Terapia Cellulare, Divisione Ematologia, Divisione Malattie Infettive, University of Genoa, San Martino Hospital Genoa, Italy

Blood stream infections (BSI) are a well-known cause of morbidity and mortality in hematopoietic stem cell transplant (HSCT) patients. The aim of this study was to analyze etiology and microbial resistance of BSI in patients undergoing allogeneic HSCT in a single center over a 4-year period (2004–2007). There were 168 episodes of BSI in 132 patients (median 10 days after HSCT) and 182 pathogens were isolated. Gram-positive bacteria (GPB) accounted for 57% of 182 isolates. Gram-negative rods (GNR) accounted for 37% and fungi for 6%. All patients received routine fluoroquinolone prophylaxis. There was a significant decrease in GPB/GNR ratio over time, from 2.4 in 2004 to 1 in 2007 (*p*=0.043). Among GPB, staphylococci decreased from 37 of 68 (64%) in 2004–2005 to 8 of 35 (23%) in 2006–2007 (*p*<0.002). The *Enterococcus faecalis*/*E. faecium* ratio decreased from 4.5 in 2004 to 0.33 in 2007 (*p*=0.006), whereas the total number of enterococcal strains per year did not change. The incidence of *Escherichia coli* among GNR increased from 3 of 15 (20%) in 2004 to 13 of 21 (62%) in 2007 (*p*=0.003). Fluoroquinolone-resistance was common, both among GPB and GNR (81% and 74%, respectively). Mortality rate at 7 days after BSI was 11% (19 of 168), reaching 39% for *Pseudomonas aeruginosa* BSI (7 of 18). BSI remains a frequent and potentially life-threatening complication of allogeneic HSCT, the causative organism influencing 7- and 30-day mortality rate. BSI etiology may change rapidly, requiring implementation of new empirical-therapy schemes.

# Sub article: Fungal infection in HSCT

**Published:** 2012-05-23

**Authors:** Shinichiro Mori

**Affiliations:** Division of Hematological Malignancy, St. Luke's International Hospital, Japan

Hematopoietic stem cell transplant (HSCT) recipients have the highest risk of acquiring invasive fungal infection (IFI) that may be associated with significant morbidity and mortality. Prevention and early recognition of IFI is crucial in improving outcomes in HSCT recipients.

HSCT recipients have a unique nature of their immunocompromised status. Profound and long-lasting neutropenia and qualitative deficits in phagocyte function during early posttransplant period (first month, in general) are among the risk factors for all kinds of fungal species infection. During this period, HSCT recipients also have mucosal damage, which allows tissue invasion of enteric fungi, principally *Candida* species.

Deficiencies of T-cell immunity arising from lack of donor-derived T-cell function, immunosuppressive agents for prevention and treatment of Graft-versus-Host disease (GvHD), GvHD itself and corticosteroid use persist for longer period (> 6 months) after HSCT. Since T-cell have a major role for protection against fungal pathogens, susceptibility for various IFIs still persists. However, as prevention and management of yeast infection improves, the peak of invasive yeast infection appears to be shifting to the later posttransplant period. Environmental control measures, such as HEPA-filtered room, and prophylactic usage of antimold agents are the mainstay of prevention. Treatment of invasive aspergillosis (IA) is also improved. Historically, mortality rate of IA among HSCT recipients have exceeded as high as 80%, although recent epidemiological studies suggest that outcomes appear to be improving. Surprisingly, a recent prospective epidemiological study conducted by a North America group (PATH Alliance) revealed that the 6-week survival rate was significantly better for HSCT recipients with IA, followed by those with invasive candidasis and those with zygomycoses or other mold.

Humoral immunity is also compromised after HSCT. Especially, recipients with chronic GvHD have long-lasting humoral immunity deficiency. Since opsonization with anticapsular antibody have a role for protecting against *Cryptococcus neoformans*, recipients with chronic GvHD and functionally splenectomized patients have high risk of acquiring cryptococcosis. However, as a result of broad usage of azole prophylaxis, cryptococcosis following HSCT seems to be very rare.

As I described above, epidemiology, morbidity and mortality of IFI after HSCT have been dramatically changing. These changes are results from improvement in prophylaxis, early diagnosis and treatment with newer antifungal agents.

In this review presentation, I would like to summarize the historical changes in IFI after HSCT and then, make it clear what are our current problems to be solved and future perspectives.

# Sub article: Infectious complications following reduced-intensity cord blood transplantation

**Published:** 2012-05-23

**Authors:** Shuichi Taniguchi

**Affiliations:** Department of Hematology, Toranomon Hospital, Tokyo, Japan

The number of UCBT has been increasing progressively, and >1000 UCBT was performed in 2010 in Japan, which is comparable to related- and unrelated bone marrow (BM) and peripheral blood (PB) transplantation. Rapid availability and less stringent HLA match requirement are the main reasons of expanding UCBT. In Japan, 40% of UCBT were performed in elderly patients (>40 years) using reduced-intensity conditioning. Relatively more urgent transplants in elderly patients and related donor unavailability due to donor's older age. UCB presents opportunities of allogeneic transplant to these aged patients.

Most serious complications in UCBT have been supposed to be infectious complications due to neutrophil and immunological recovery delay compared to BM and PB stem cell transplantation. Actually, neutrophils recovery delays by almost 7 days after UCBT, which might increase the incidence of severe bacterial infection. Immunological recovery, especially antigen-specific cytotoxic T-lymphocyte (CTL), has been reported to delay significantly in UCBT compared to PB and BM. Various viral infections occur more frequently and severely. Neutrophil recovery delays mainly due to low total cell and CD34^+^ number in cord blood, and immunological recovery delay was explained by dominant naïve T cells in transfused cord blood.

We have been involved in more than 700 UCBT from 2003 to 2011. Our patients are relatively older patients (mean age, 58–59 years) with advanced disease (80%). For these reasons, reduced-intensity conditioning was used in majority of UCBT (90%). In this case cohort, we analyzed T cell and B cell recovery (Fig. 1) and immunoglobulin production in CBT and compared with PB and BM patients. T cell subset recovery was relatively rapid and B and NK cell tend to recover earlier than unrelated BM. Immunoglobulin supplement to maintain IgG above 500 mg/dl was compared among related PB, unrelated BM and UCB. Although the onset of chronic graft-versus-host disease (cGVHD) and intensification of immunosuppression are the major determinant of IG production, IG supplement was less and shorter after transplant in UCB group compared to RPB and UBM group.

We also analyzed the incidence and species of bacteremia, viral infection (CMV, HHV6, EBV and ADV) and fungal infection. In symposium, I will discuss these results.

**Fig. 1:** Immune recovery after CBT adult in patients.

# Sub article: Contribution of intestinal flora to homeostasis and ibd pathogenesis

**Published:** 2012-05-23

**Authors:** Toshifumi Hibi, Tadakazu Hisamatsu, Takanori Kanai

**Affiliations:** Division of Gastroenterology and Hepatology, Department of Internal Medicine, Keio University School of Medicine, Tokyo, Japan

Inflammatory bowel disease (IBD) is classified into two typical phenotypes, ulcerative colitis and Crohn's disease. Although the precise etiologies of IBD remain unclear, several studies have indicated that dysfunction of the mucosal immune system plays important roles in its pathogenesis. Especially, recent studies spotlight the cross talk between host and intestinal flora. The gut has 10^10–12^ bacteria through entire digestive tract. It means that gut is always exposed to enteric bacteria and food antigens; however, gut maintains its homeostasis without development of chronic inflammation in normal situation. Recent studies have demonstrated that (1) gut microbiota may contribute to gut immunological homeostasis, (2) gut has protective mechanisms such as intestinal macrophages system to suppress excess immune response to those foreign antigens and (3) the disruption of those regulatory systems may cause IBD.

One of the most important concepts of IBD pathophysiology is that the homeostasis of gut immune system to enteric flora becomes discordant. Intestinal macrophage is a key player for not only elimination of bacteria by phagocytosis but also intestinal immune homeostasis. We have revealed a functional role of intestinal macrophages for gut homeostasis and the fact that disregulation of intestinal macrophages to commensals lead to chronic intestinal inflammation in Crohn's disease.

Crohn's disease (1) is characterized by the Th1 dominant chronic intestinal inflammation. We identified that number of CD14^+^CD33^+^CD68^+^ unique intestinal macrophages were increased in lamina propria (LP) in the patients with IBD, especially Crohn's disease. These cells showed typical macrophages morphology, but they expressed some DC markers and they had antigen-presenting function. CD14^+^ intestinal macrophages induced both Th1 and Th17 cells from peripheral blood naïve T cells. Intestinal bacteria enhanced Th17 polarization through IL-1β and IL-6 produced by CD14^+^ intestinal macrophages, while IL-23 enhanced Th1 immunity. Thus, CD14^+^ intestinal macrophages may be involved in the pathogenesis of Crohn's disease as antigen presenting cells (APCs) (2).

In local immunity, these intestinal Mfs produce large amount of TNFa and IL-23, which are key cytokines for Crohn's disease pathogenesis, in response to commensal bacteria. IL-23 enhanced production of IFNγ by LP mononuclear cells. We identified that the source of IFNγ are CD4, CD8 T cells and mucosal natural killer cells. In addition, TL1A cooperating IL-23 may synergistically enhance IFNγ and IL-17 production by LP CD4^+^ T cells (3).

In conclusion, CD14+ intestinal macrophages play the central roles in the pathogenesis of Crohn's disease by regulating local immunity and inducing both Th1 and Th17 immunity by APCs.

## References

[1] Kamada N, Hisamatsu T, Hibi T (2009). Retinoic acid contributes to the induction of IL-12-hypoproducing dendritic cells. Immunol.

[2] Kamada N, Hisamatsu T, Hibi T, Kobayashi T, Chinen H, Kitazume MT (2009). Human CD14+ macrophages in intestinal lamina propria exhibit potent antigen-presenting ability. J Immunol.

[3] Kamada N, Hisamatsu T, Hibi T, Chinen H, Kobayashi T, Sato T (2008). Unique CD14 intestinal macrophages contribute to the pathogenesis of Crohn disease via IL-23/IFN-gamma axis. J Clin Invest.

# Sub article: Mucosal decisions for immunity to co-habitation of microflora

**Published:** 2012-05-23

**Authors:** Hiroshi Kiyono

**Affiliations:** Division of Mucosal Immunology, The Institute of Medical Science, The University of Tokyo, Tokyo, Japan

Digestive tract is covered by a single layer of mucosal epithelial cells, which is continuously exposed to infinite antigenic challenges in handling its day-to-day duties. The intestinal tract is thus equipped with the mucosal immune system (MIS) offering the first line of innate and acquired defense forces against invasion of pathogens and, hence, required to induce a prompt and robust immune response in order to prevent invasion of infectious agents. At the same time, the MIS also exposed to an enormous number and volume of innocuous and/or instructive antigens, which need to be appropriately ‘ignored’. Mounting an immunologically harmonized response, therefore, represents a key decision-making process of active and/or quiescent immune responses by the MIS. The mucosal surface covering the digestive tract represents a complex immunological network structured to execute the immunologically harmonized regulation of the two opposite immune responses. For the understanding of the harmonized MIS, it is essential to elucidate the molecular and cellular mechanisms of crosstalk system between the MIS and commensal flora. Our recent results suggested that gut-associated lymphoid tissues (GALT) including Peyer's patches play a critical role in the creation of cohabitation niche between the host and commensal bacteria. Our recent studies have now thus provided new evidence for the intratissue habitation of commensal flora in the organized lymphoid structure associated with mucosa (e.g., GALT).

# Sub article: Control of immune responses by commensal bacteria during acute gastrointestinal infections

**Published:** 2012-05-23

**Authors:** Liliane M. dos Santos, Timothy Hand, Nicolas Bouladoux, Yasmine Belkaid

**Affiliations:** 1 Laboratory of Gnotobiology and Immunology, Federal University of Minas Gerais, Brazil; 2 Laboratory of Parasitic Diseases, National Institutes of Health, USA

The gastrointestinal tract of mammals is inhabited by thousands of distinct species of commensal microorganisms that exist in a mutualistic relationship with the host. It has previously been shown that these gut microbes play an important role in modulating host immune responses. On the other hand, commensals can also contribute to pathology in the context of acute infection. For instance, oral infections with *Toxoplasma gondii* in certain inbred strains of mice lead to an exacerbated intestinal inflammation that is accompanied by a loss of diversity within the gut flora. Furthermore, the microbiota was shown to aggravate the immunopathology of the disease. The mechanisms underlying this phenomenon still remain poorly understood. In order to study how the recognition of innocuous microbes can influence immune responses and pathological consequences during acute mucosal infections, we treated mice with a cocktail of antibiotics. Antibiotic-treated mice showed decreased inflammatory responses and lower parasite load. Studies using germfree mice were also carried out. Germfree mice infected with *T. gondii* displayed less severe pathology and reduced parasite burden. Dysruption of intestinal homeostasis during *T. gondii* infection led to systemic translocation of gut bacteria and temporal changes in the diversity of the gut microbial community. Three different bacteria that were abundant in the gut of *T. gondii-*infected mice at the peak of infection were isolated and used for investigation of specific immune responses against commensal bacteria. We showed that *T. gondii* acute infection induces specific antibody responses toward antigens from the microbiota.

# Sub article: Physiological role of indigenous Lactobacilli/*Helicobacter pylori* in the stomach

**Published:** 2012-05-23

**Authors:** Yasuhiro Koga

**Affiliations:** Laboratory for Infectious Diseases, Tokai University School of Medicine, Isehara, Japan

The intestinal tract harbors a rich variety of microbiota consisting of hundreds of different bacterial species containing high densities of living bacteria, which achieve concentrations of up to 10^11^ or 10^12^ cells/g of luminal contents. The role of such indigenous microbiota of the gut in health and disease is well known to include metabolic activities, trophic effects on intestinal epithelia and the immune system and protection of the colonized host against invasion by alien microbes.

On the other hand, the stomach contains only a few species of bacteria in the human if it is free from infection with *Helicobacter pylori*. During fasting, the gastric juice contains only small numbers of bacteria, approximately, 10^2^ to 10^3^/ml, which include *Streptococcus*, *Lactobacillus* and *Veillonella*. However, these bacteria are considered nonresidents that are just in transit from the oral cavity and throat. The scarcity of such bacteria in the human stomach appears to be because of the high acidity of the luminal medium.

While *H. pylori* is a well-known pathogenic bacterium that causes peptic ulcers and cancer in the human stomach, *Helicobacter* species have also been proposed to belong to the indigenous gastric microbiota of humans from our earliest times. That hypothesis is supported by the fact that *H. pylori* is acquired in early childhood and thereafter remains stably colonized in the stomach for decades in substantial numbers. This raises questions about the role of the indigenous bacteria of the stomach in the physiological development and function of this organ. However, it is difficult to clarify the answers to this question by an infection study using *H. pylori*, because various pathogenic factors of *H. pylori* such as CagA, vacuolating toxins, urease and its metabolites induce chronic pathological inflammation in the gastric tissue, which thus obscures the physiological role of *H. pylori* as an indigenous bacterium.

In a previous study, we found an indigenous microbiota, which predominantly consists of lactobacilli, in the stomach of specific pathogen-free mice. The lower acidity in the stomach of mice was thought to enable the lactobacilli to colonize the stomach. Moreover, no evident inflammatory changes occurred in the stomach of the mice. In a recent study, a microarray analysis was performed to investigate the role of these innate lactobacilli in the development of physiological functions of the stomach using germfree (GF) and lactobacilli-associated gnotobiotic mice.

In this DNA microarray analysis, GF BALB/c mice were orally inoculated with 10^9^ CFU lactobacilli and their stomachs were excised after 10 days to extract RNA. As a result, lactobacilli-associated gnotobiotic mice showed a dramatically decreased expression of the gastrin gene in comparison to germfree mice. The mean of the log_2_ fold change of gastrin gene was −4.3. Immunohistochemistry also demonstrated the number of gastrin^+^ cells to be significantly lower in the lactobacilli-associated gnotobiotic than in the GF mice. Moreover, oral inoculation of heat-killed lactobacilli to GF mice also decreased the gastrin^+^ cell number. However, there was no significant difference in the number of somatostatin^+^ cells in these groups of mice. Consequently, gastric acid secretion also decreased in the mice colonized by lactobacilli. While an increase in the expression of the genes related to the muscle system development, such as nebulin and troponin, was observed in lactobacilli-associated mice too, either the mechanism of increase or the relevance to the suppression of gastrin remains to be obscured. Taken together, these results suggested that indigenous lactobacilli in the stomach significantly affect the regulation of gastrin-mediated gastric acid secretion without affecting somatostatin secretion in mice.

# Sub article: Development of novel methods for the search of antibiofilm agents

**Published:** 2012-05-23

**Authors:** Reiko Kariyama, Hiromi Kumon

**Affiliations:** Department of Urology, Graduate School of Medicine, Dentistry and Pharmaceutical Sciences, Okayama University, Okayama, Japan

Over the last 20 years, we have been investigating biofilm infections in the urinary tract. For the prevention and control of biofilm infections, many basic and clinical studies are currently in progress worldwide. The important step in studying biofilm infections is establishing good experimental models and methods of evaluation. It is necessary to make a major breakthrough toward the development of novel therapies, medical devices and innovative antibiofilm agents. In this presentation, our recent experimental approaches by using real-time imaging *in vitro* and *in vivo* models to search antibiofilm agents are reviewed.

A capillary flow cell system as an *in vitro* model of complicated urinary tract infections (UTI) is utilized. *Pseudomonas aeruginosa* OP14-210 isolated from a patient with catheter-associated UTI was used and a green fluorescent protein (GFP)-producing strain, *P. aeruginosa* OP14-210 (pMF230), was constructed. Biofilms were grown in glass capillary tubes under continuous flow conditions with artificial urine and were observed by confocal laser scanning microscopy. To evaluate the effects of potential antibiofilm agents, levofloxacin (LVFX 10 times the MIC: 80 µg/ml), ulifloxacin (UFX 10 times the MIC: 20 µg/ml) and fosfomycin (FOM 3 times the MIC: 192 µg/ml) were tested. When both LVFX and FOM were added to the system 2-h after inoculation with the GFP-producing strain, very weak fluorescence signal indicating no biofilm formation was observed after 3 days. The GFP-producing 1-day biofilm after 72-h treatment with FOM alone was similar to that seen with no treatment. The irregular detached biofilm was observed by the treatment with LVFX alone. In combination of LVFX and FOM, the irregular detached biofilm was much thinner than that with LVFX alone. BacLight staining was applied to assess the effects of treatment on the number of live and dead cells and their distribution in biofilms. A higher proportion of dead cells was observed in the 2-day biofilms after 18-h treatment with either UFX alone or in combination of UFX and FOM compared with either LVFX alone or in combination of LVFX and FOM. The quantitative analysis of the intensity of green and red signals confirmed the increased bactericidal effect by the combination of UFX and FOM compared with LVFX and FOM.

More recently, we started to develop a new type of microdevice to screen antibiofilm agents efficiently. We designed the new type of microdevice, which might be set on microscope stages and evaluated the effects of many samples simultaneously. The specification of a model of microdevice with porous media is a double structure with a layer of biofilm formation and a layer of drug supply. The specification of a model of microdevice without porous media is three-step slopes, which are able to observe changes in biofilm phenotype by a different flow rate. Our previous findings by using a capillary flow cell system regarding the synergy between LVFX and FOM were confirmed using the new type of microdevice (the latest model without porous media). The basic design of a new type of microdevice for efficient screening of antibiofilm agents was almost established by continuous improvements. By using the new type of microdevice, it is possible to screen novel compounds and the combination of possible antibiofilm agents for the treatment of biofilm infections.

On the other hand, we assessed a noninvasive, real-time imaging technology (IVIS^®^ imaging system, Caliper Life Sciences) for animal models of *P. aeruginosa* biofilms in UTIs. To establish animal models, SD rats (female, 7 to 8 weeks old) and ICR mice (female, 6 to 7 weeks old) were used. According to the previous report (1), a spiral-shaped polyethylene tube (PT) was placed as a foreign body in the rat bladder without surgery. The bioluminescent *P. aeruginosa* Xen 5 and Xen 41 and GFP-producing *P. aeruginosa* OP14-210 (pMF230) were used. One week after inoculation, bioluminescence images were detected when enough urine was kept in the rat bladder, but biofilms on spiral PTs were not detected. In a novel procedure, two PTs were placed into the mouse bladder. We have established the rat and mouse models of *P. aeruginosa* biofilms in UTIs. However, we are not able to detect bioluminescence and fluorescence images of biofilms on spiral PTs *in vivo* by using the IVIS^®^ Lumina system. It was thought that this problem might be addressed by using novel bioluminescent strains engineered.

Most recently, we have found that a quorum sensing inhibitor of *P. aeruginosa* would be a strong anti-*Pseudomonas* agent combined with biapenem in a murine (male, 5 to 6 weeks old) neutropenic thigh infection model of a bioluminescent *P. aeruginosa* Xen 5 strain by using the IVIS^®^ Lumina system. We confirmed that biapenem exhibits an antimicrobial effect with the time above MIC (T>MIC) in pharmacokinetic and pharmacodynamic (PK/PD) parameters. We also demonstrated a good correlation between photon count imaging and viable counts *in vivo*. By using *in vitro* models described above, the quorum sensing inhibitor of *P. aeruginosa* was found to inhibit biofilm formation of *P. aeruginosa*. Our experiments intended to establish the applicability of some animal models in efficacy assessments of therapeutic agents for treatment of chronic infections by bacterial biofilms in the future.

## References

[1] Kurosaka Y, Ishida Y, Yamamura E, Takase H, Otani T, Kumon H (2001). A non-surgical rat model of foreign body-associated urinary tract infection with *Pseudomonas aeruginosa*. Microbiol Immunol.

# Sub article: Two-component signal transduction systems and biofilm formation of *Staphylococcus epidermidis*

**Published:** 2012-05-23

**Authors:** Di Qu, Yang Wu, Shiqing Han, Xu Shen, Hualiang Jiang

**Affiliations:** 1 Key Laboratory of Medical Molecular Virology of Ministries of Education and Health, Institute of Medical Microbiology and Institutes of Biomedical Sciences, Shanghai Medical College of Fudan University, Shanghai, China; 2 College of Biotechnology and Pharmaceutical Engineering, Nanjing University of Technology, Nanjing, China; 3 Drug Discovery and Design Center, State Key Laboratory of Drug Research, Shanghai Institute of Materia Medica, Chinese Academy of Sciences, Shanghai, China

Coagulase-negative *Staphylococcus epidermidis* has emerged as one of the most important nosocomial pathogens. The pathogenicity of *S. epidermidis* is mostly due to its ability to form a thick, multilayered biofilm on polymeric surfaces of implanted medical devices. Treatment of *S. epidermidis* infection has become a troublesome problem as biofilm-associated bacteria exhibit enhanced resistance to antibiotics and to components of the innate host defense. Based on the two complete genome sequences of *S. epidermidis* (ADCC12228 and ADCC35984), we have analyzed two-component signal transduction systems (TCSs) in *S. epidermidis*, which consists of 16 or 17 pairs TCSs. Each TCSs consists of a signal ligand responsive histidine kinase (HK) and a response regulator transcription factor present in bacteria. Some of them have been identified to regulate diverse functions of bacteria including biofilm formation, virulence, cell wall biosynthesis, quorum sensing, etc. Our research is mainly focused on the roles of TCSs in *S. epidermidis* biofilm formation. We have investigated the effects of *lytSR*, *saeRS*, *arlRS* and *srrBA* knocking out on biofilm formation and their regulated genes. We found that biofilm formation was enhanced in *ΔlytSR*, *ΔsaeRS* strains, while decreased in *ΔarlRS* and *ΔsrrBA* strains. The mechanisms of *lytSR*, *saeRS*, *arlRS* and *srrBA* in regulation of biofilm formation are under studying.

In another aspect, we are trying to discover inhibitors targeting to HK domain in YycG protein of *S. epidermidis*. Using structure-based virtual screening (SBVS) from a small molecular lead-compound library, followed by experimental validation, the inhibitors of YycG that we discovered displayed bactericidal effects on both planktonic and biofilm cells of *S. epidermidis*. To improve the inhibition and bactericidal activities of one of compounds (compound 2) on *S. epidermidis* biofilm, a series of the derivatives were synthesized by cyclization, aldol condensation, substitution and hydrolization reactions. The six derivatives out of 46 synthesized new compounds inhibited phosphoryl transfer activity of YycG histidine kinase and were proven to kill bacteria in both immature and mature biofilms of *S. epidermidis* more effectively than the leading compound. However, the structures of those YycG inhibitors need to be improved for increasing the potential application as antibiofilms agents.

# Sub article: Biofilm formation of *Helicobacter pylori*

**Published:** 2012-05-23

**Authors:** Hideo Yonezawa, Shigeru Kamiya

**Affiliations:** Department of Infectious Disease, Kyorin University School of Medicine, Tokyo, Japan

Biofilms are surface-bound communities of bacterial cells that are implicated in their survival. Recently, some reports have indicated that *Helicobacter pylori* forms biofilm in vitro and in vivo; however, biofilms of *H. pylori* have not been well characterized. We attempted to analyze the ability of *H. pylori* strains to form biofilms in vitro and characterized the underlying mechanisms of *H. pylori* biofilm formation in Brucella broth supplemented with 7% fetal calf serum (FCS). Strain TK1402 showed strong biofilm forming ability relative to the other strains under these conditions. There were no significant differences in the aggregation, motility and hydrophobicity of strain TK1402 compared with the other strains. The strong biofilm forming ability of TK1402 reflected the thickness of the biofilms. In addition, the biofilm formation was correlated strongly with the production of outer membrane vesicle (OMV) in strain TK1402. SEM analysis indicated that OMV was detected within the matrix of only the TK1402 biofilms. Strain TK1402 did not form thick biofilms in Brucella broth supplemented with 0.2% β-cyclodextrin; however, the addition of the OMV-fraction collected from TK1402 could enhance biofilm formation. Taken together, these results suggest that the TK1402 is a strongly biofilm-forming strain in Brucella broth supplemented with 7% FCS, and the OMV production from this strain play an important role in biofilm formation.

Next, we analyzed the comparison of the urease production between biofilm cells and planktonic cell. In Western blotting analysis and quantitative RT-PCR with TK1402 biofilm cells, the level of the urease expression was increased in biofilm cells compared to planktonic cells. These suggested that the biofilm formation might be associated with virulence of this microorganism.

# Sub article: Immune response of the gnotobiotic mouse model induced mycoplasmal pneumonia and evaluation of antimicrobials using the model

**Published:** 2012-05-23

**Authors:** Haruhiko Taguchi, Satoshi Kurata, Ken Arae, Tsuyoshi Onogawa, Shigeru Kamiya

**Affiliations:** 1 Department of Immunology, Kyorin University Faculty of Health Sciences, Tokyo, Japan; 2 Department of Infectious Diseases, Kyorin University School of Medicine, Tokyo, Japan

Mycoplasmas are the smallest and self-replicating microorganisms. They are primarily mucosal pathogens, residing with epithelial surfaces extracellularly. *Mycoplasma pneumoniae* is an atypical bacterium that lacks a cell wall and is recognized as an important etiologic agent of acute lower respiratory infection in children and young adult. In Japan, approximately 30% to 40% of community-acquired pneumonia cases are due to *M. pneumonia* infection. Mycoplasmal pneumonia is considered to be a relatively benign disease that is improved by appropriate treatment with antimicrobials in many cases, even though it presents symptoms such as high fever, persistent cough and dyspnea. However, in some cases, it becomes severe by presenting various clinical features such as the development of bronchial asthma and complication by extrapulmonary lesions. Histopathologically, in mycoplasmal pneumonia, the bronchial and bronchiolar lumina are characteristically filled with polymorphonuclear leukocytes, and their walls have a mononuclear infiltration with plasma cells.

Although the mechanism by which primary atypical pneumonia is caused by *M. pneumoniae* has not been clarified, it is hypothesized that indirect injury through immune responses of the host besides the direct pathogenic factors of *M. pneumoniae* is important in the infection. Several animal models have been developed to examine the pathogenesis of mycoplasmal pneumonia; however, the importance of host immune response related to the pneumonia is not revealed. The traditional animal model of mycoplasmal pneumonia, the Syrian hamster, is limited by an inability to study the host cytokine response due to a lack of host-specific reagents. Thus, establishment of a murine model is extremely important to help elucidate the host immune response to *M. pneumoniae* infection and to better understand the pathogenesis of mycoplasmal pneumonia. On the other hand, gnotobiotic animal offer a well-defined model to study the pathogenicity of bacteria because it is possible to study the interaction of the bacterium with the host without the influence of any other bacteria. So, we attempted to establish an animal model using germfree mice for *M. pneumoniae* infection, and the immunological, histopathological and bacteriological studies were performed.

In this presentation, we would like to show the host immune response of the gnotobiotic mouse model induced mycoplasmal pneumonia and the efficacy of some anti-*M. pneumoniae* drug possessing immunomodulation activity independent of its antimicrobial activity.

# Sub article: Epidemiology, diagnosis, medication and vaccination of avian mycoplasmosis in Taiwan

**Published:** 2012-05-23

**Authors:** Michael M.Y. Lin

**Affiliations:** Department of Veterinary Medicine, College of Veterinary Medicine, National Pingtung University of Science and Technology, Neipu, Pingtung, Taiwan

Cultivation of variant serotypes of avian mycoplasmas with Frey's medium from the tracheal swabs of chickens, ducks, geese and English sparrows in Taiwan during 1985–1995 provided 47.8%, 5.6%, 18.6% and 0.03% positive of isolation, respectively. Most of the poultry flocks in Taiwan were infected with mycoplasmas.

Rapid identification of variant serotypes of avian mycoplasmas using nested and serotype-specific polymerase chain reaction (PCR) for amplification of the rRNA gene and identification of the serotype relevant sizes of generated amplicons under agarose gel electrophoresis was established in this laboratory since 2001. Molecular identification of avian mycoplasmas comes to be more scientific than used to be done by fluorescent antibody technique.

Electrophoresis and immunoelectrophoresis of variant serotypes and strains of avian mycoplasma cellular and membrane proteins provided that different serotype or strain of avian mycoplasmas has its unique pattern of cellular or membrane protein profile, which can be used for serotyping or strain characterization.

Simple immunodot system (SIM) implying the reading of simple immunodot blotting assay (SIDBA) by Amersham computer software system was used to evaluate the intensity of the dot density in numerical scale. SIM assay provided high sensitivity and moderated specificity in the detection of MG antibody in chickens.

Inhibitory activity of all the commercial available antimicrobials against the standard strains and the 1987, 1994 and 1999 Taiwanese isolates of avian mycoplasmas had been evaluated in this laboratory. Most of the mycoplasmas were found with gradually developing their resistance against the antimicrobials recently used in the poultry farms in Taiwan. Only around 10 commercial antimicrobials were found with the MIC_90_ level <10 µg/mL that are good to be used in the poultry clinic for treatment of avian mycoplasmosis in Taiwan.

Evaluation of five attenuated strains of MG as live vaccine in young chickens. Five trials were conducted to evaluate the virulence and the vaccination efficacy of the F, R, S6 and A5969 strains of MG at different in vitro passage levels. Vaccination was done by eye-drop or aerosol, and the efficacy was evaluated in terms of air-sac lesion scoring level after aerosol challenge with the R strain of MG. Continuing the medium passage of these strains of MG resulted in gradual attenuation. Aerosol vaccination with highly attenuated MG at 21 days of age was more effective in stimulating the development of immunity than was eye-drop vaccination at 7 days but inducing higher air-sac lesions. Aerosol vaccination with high-passaged F or S6 strain provided good immunity against MG challenge. Both low- and high-passaged (250p) F and S6 strains were found still virulent for turkey poults, but only the high-passaged F strain significantly (*p*<0.05) less affected the body weight gaining. The MG-F strain was finally chosen as live vaccine production strain. The MG-F strain low passage has been marketed worldwide by Schering-Plough Co. Ltd. for more than 10 years. The MG-F strain high passage has been intended to be introduced in the market.

Field application of MG-F strain live vaccine in Taiwan. MG-F strain live vaccine has been tried with only good for applying in leghorn layers but not good for broilers, native hybrid broilers or any kind of broiler breeders. Most of the layers originated from mycoplasma-free breeder flocks were supposed to be vaccinated at 10 to 15 weeks of age. Most of the layers originated from mycoplasma-infected breeder flocks were supposed to be vaccinated at 2–3 weeks of age. All the chicks subjected to be vaccinated supposed to be treated with some antibiotic (tiamutin, etc.) for 3 days first and then skipped off any antibiotic treatment at least for 7 days before vaccination. MG-F strain for eye drop vaccination shall be under 30th passage level, for aerosol vaccination shall be at around 250th passage level. Eye drop vaccination of MG-F strain can be done in combination with ND and IB live vaccines simultaneously using MG vaccine as diluent.

Preparation of MG-R strain bacterin and its application in broiler breeder in Taiwan. Hybrid broiler breeders were intramuscularly immunized (0.5 ml/ml) twice with MG-R bacterin at 7 and 15 weeks of age. MG-R bacterin provided some improved egg production (4.5 more egg production during first 82 days laying in comparison with the nonimmunized control.

# Sub article: Diagnosis of *Mycoplasma pneumoniae* and other respiratory tract infections using gene amplification method ‘loop-mediated isothermal amplification (LAMP)’

**Published:** 2012-05-23

**Authors:** Yoshinori Ota, Manabu Yoshino, Toshimitus Annaka, Tsugunori Notomi, Hidetoshi Kanda

**Affiliations:** Biochemical Research Laboratory, Research & Development Division, Eiken Chemical Co., Ltd., Tochigi, Japan

There are several kind of bacteria that are frequently isolated as the causative pathogens of community-acquired pneumonia, for example, *Streptococcus pneumoniae*, *Haemophilus influenzae* as well as atypical pneumonia pathogen *Mycoplasma pneumoniae*, *Chlamydia pneumoniae* and *Legionella* spp.

Especially, the pneumonia caused by *M. pneumoniae* or *Legionella* spp. sometimes increases in severity. Since a delay in an early treatment is associated with increased mortality, an appropriate therapy of these pneumoniae must be done immediately. However, beta-lactam antibiotic, which is usually a first choice for bacterial pneumoniae treatment, does not work against these atypcial pathogens. For an effective treatment, it is significantly important to choose the antibiotics based on a judgement whether a patient is suffering from the bacterial pneumonia or the atypical pneumonia.

For an identification of these causative bacteria, the most reliable method is a culture test. But the method requires a specail technique for its operation, and further, it takes a longer period to obtain the results, which will not be used as a guide for an early treatment but just a diagnosis after the treamtment. Due to those reasons, a relatively small number of the culture test has been conducted as an ordinary clinical laboratory test.

In order to provide a solution to these problem, we developed a nucleotic acid tests for a detection of *M. pneumoniae* and *Legionella* spp., using LAMP method, and studied its clinical usefulness.

LAMP, which was invented by Eiken Chemical Co., Ltd. in 2000, is the nucleic acid amplification technique that rapidely amplifies and detects DNA. The LAMP method employs four unique primers and DNA polymerase possessing the strand-displacement activity, which enables a consecutive DNA amplification reaction under an isothermal condition (60–65°).

The LAMP reaction proceeds through a repetion of both a self primed elongation, which initiates from a stem-loop structure of an amplified product, and a strand-displacement DNA sysnthesis, which starts from inner primer annealing to a loop structure portion of the amplicon.

Because LAMP method runs consective gene amplification reaction, its gene amplification efficiency is enormousely high, which allows for a rapid amplificatin of a target gene (<1 h.). Its reaction can be monitored by means of a turbidity caused by a large amount of by-product generated during the gene amplification reaction, which enables a single tube reaction of whole process from a gene amplification to a detection.

For our clincal diagnosis kits, we used *SDC* gene, which is a part of the repeted gene sequences involved in host cell adhession, as target site for the detection of *M. pneumoniae* and 16S rDNA for *Legionell* spp.

The clinical study of our kits showed a good agreement with a those of the conventional method as well as PCR. We concluded that our kits are the useful tools to identify *M. pneumoniae* or *Legionella* spp., differentiate them from other respiratory tract infections and help medical treatment.

Further, we will introduce our approach to a Point of Care Testing using LAMP method that makes the nucleic acid test for other respiratory tract infections (*Mycobacterium tuberculosis* or influenza virus) more feasible in a usual clinical laboratory.

# Sub article: Autologous hematopoietic stem cell preservation in nuclear plant workers

**Published:** 2012-05-23

**Authors:** Shuichi Taniguchi

**Affiliations:** Department of Hematology, Toranomon Hospital, Tokyo, Japan

The 9.0 magnitude earthquake and following tsunami on March 11, 2011, destroyed many coastal cities in the northeastern part of Japan. It swamped emergency generators at the Fukushima Daiichi nuclear power plant operated by the Tokyo Electric Power Company in Fukushima prefecture, disabling the cooling systems. Since these catastrophic events, hundreds of nuclear workers have been trying to remove the radioactive water from the tsunami ravaged nuclear compound and restart the regular cooling systems for the overheated nuclear fuel. On April 12, the Nuclear and Industrial Safety Agency of Japan decided to raise the severity level of the crisis to 7—the highest level and equal to the 1986 disaster at Chernobyl in the former Soviet Union. We emphasize the need to predict potential scenarios in Fukushima and to prepare medical care providers for how to respond in cases of accidental high radiation exposure, since this operation is estimated to take months to years.

Generally, rapidly dividing cells, such as intestinal-tract and hemopoietic cells, are most vulnerable to radiation. Radiation accidents can result in localized or whole-body exposure and in internal or external deposition of radioactive materials. On March 24, three workers at the Fukushima nuclear power plant were exposed accidentally to high localized radiation while standing in contaminated water. Fortunately, this accident did not cause major injuries, but the danger of a future accidental radiation exposure is not passed, since there has been a series of serious aftershocks even this April.

A clinically significant hemopoietic syndrome can occur after whole body doses of 2 Gy or higher as a result of bone-marrow depression. If the hemopoietic cells are not completely damaged, a recovery phase can be enhanced through the use of hemopoietic growth factors. In cases of radiation exposure of more than 5 Gy, hemopoietic stem cell rescue is essential. Indeed, in the Chernobyl disaster in 1986, nine patients who were exposed to high levels of radiation underwent intraosseous injections of allogeneic bone-marrow cells. In the radiation accident at a nuclear fuel processing plant in Tokaimura, Japan, in 1999, two victims received allogeneic stem-cell transplantation. However, allogeneic stem-cell transplantation has major limitations, such as time consuming donor searching, graft failure, graft-versus-host disease (GVHD) or profound immune suppression after transplantation, despite the reduced mortality associated with recent techniques.

On March 25, we proposed the collection and storage of autologous peripheral-blood stem cells (PBSCs) for the nuclear workers in Fukushima in case of accidental major radiation exposure. This scheme has several advantages. First, autologous PBSC transplantation does not cause GVHD, which further exacerbates gut injury mediated by radiation exposure. Second, it does not require immunosuppressants, which make radiation victims more susceptible to severe infections. Third, PBSCs can induce more rapid hemopoietic recovery than can hemopoietic growth-factor support alone or bone marrow cells. Fourth, they are easy to store by cryopreservation. Fifth, the short-term and long-term safety of this PBSC-collecting procedure has been confirmed in a large number of healthy donors for patients with hematological cancers. Finally, long-term autologous PBSC banking might also have a therapeutic role for possible leukemia in future, because radiation is a well-known carcinogen in the long term. Several important limitations of this scheme should also be noted. Autologous PBSC transplantation is not a perfect strategy to treat radiation victims; it can rescue injury of bone marrow only, but not other tissues, such as gastrointestinal tract, skin, or lung. Additionally, there are adverse events for healthy individuals by administration of mobilizing agents and apheresis procedures. Another concern must be medical costs; but several pharmaceutical companies offer donations for this scheme and the financial burden is alleviated.

On March 29, the Japan Society for Hematopoietic Cell Transplantation released a statement that “107 transplant teams are standing by to collect and store hemopoietic stem cells from the peripheral blood for workers who are striving to restrain the radiation”. The European Group for Blood and Marrow Transplantation also announced that this plan makes sense and more than 50 hospitals in Europe have agreed to help the workers if required. The process to completely shut down the reactors in Fukushima is expected to take years. The risk of accidental radiation exposure will thus accumulate for the nuclear workers and banking of their autologous PBSCs will become increasingly important. A judgment of right or wrong on this scheme must be determined from the standpoint of the nuclear workers and their families, not from a point of view of cost-benefit balance in ordinary times. Toranomon Hospital in Tokyo is ready to harvest and bank autologous PBSCs for the nuclear workers upon request.

# Sub article: Enterococci as probiotics or autoprobiotics in treatment of the gastrointestinal diseases

**Published:** 2012-05-23

**Authors:** Alexander Suvorov, Vladimir Simanenkov, Elena Ermolenko, Viktoria Kolodjieva, Anna Tcapieva, Natalia Zaharova, Olga Solovieva

**Affiliations:** 1 Institute of Experimental Medicine RAMS, St. Petersburg, Russia; 2 Medical Academy of Postgraduate Studies, St. Petersburg, Russia

## Objectives

Enterococci are lactic acid bacteria which can be both the cause of serious infection diseases and the potent and clinically effective probiotics. The goal of the study was to analyze some differences between clinical and probiotic strains and to evaluate the possibility of usage of enterococcal indigenous strains as autoprobiotics.

## Methods

Probiotic strain *Enterococcus faecium* L3 (L3) was analyzed employing various molecular biological approaches and compared with the strains causing enterococcal infections. Autoprobiotics were isolated from human feces, tested for virulence genes and used for making milk fermented product.

## Results

Probiotic strain L3 in comparison with the clinical strains was free from virulence factor genes and carried the genes of two potent bacteriocins (enterocins A and B), which could be induced by the pheromone. Pheromone induction had been proved employing synthetic pheromones.

In addition, L3 was able to induce IL10 and decrease IL8 production in the epithelium. High antibacterial activity of L3 allowed the treatment of *Helicobacter pylori*-associated gastritis. Genomic comparison of the strain L3 with the clinical strain showed significant differences in size and genome organization. L3 strain prepared as milk fermented product was tested for the treatment of patients with IBS in comparison with autoprobiotic enterococci. Both types of bacteria (autoprobiotics and L3) showed significant improvement of the condition of the patients in double blind placebo controlled study.

## Conclusion

Probiotic enterococci and enterococcal autoprobiotics have a good potential for the treatment of gastrointestinal disorders.

# Sub article: Host/bacteria interaction and influence of beneficial bacteria on mucosal immune responses against opportunistic/enteric pathogens: possible cellular-molecular mechanisms and practical approaches

**Published:** 2012-05-23

**Authors:** Nadiiya Volodymyrivna Boyko

**Affiliations:** Uzhhorod National University, Faculty of Medicine, Uzhhorod, Ukraine

Until recently, identification and application of probiotic microbes had been serendipitous and without a sound scientific basis for selection. This was largely due to our not yet having a broad understanding of the mechanisms and effects operative upon probiotic microbe/host interactions. Clearly, some probiotic candidates have proven to be effective in increasing human and farm animal resistance to frank, nosocomial and opportunistic pathogens. The use of germfree or gnotobiotic mice should provide a set of models that will be informative about possible beneficial microbial stimulation of elements of the immune system generally classified as being at the intersection of the innate and specific adaptive immune responses: ‘natural antibodies’, whose expression may be stimulated polyclonally or oligoclonally by microbial ‘mitogens’ and/or TI-1 and TI-2 antigens; ‘natural’ killer cells and subsets of T cells that may be polyclonally stimulated via toll-like receptors or costimulated in this fashion along with microbial antigens. We believe that the effects caused by the above mechanisms may be associated with resistance to microbial translocation, colonization and pathogenesis following subsequent challenge with pathogens. We have actively pursued in detail beneficial mechanisms should our model systems provide appropriate clues.

Our initial *in vivo* analysis of humoral and cellular mechanisms by which different commensal bacteria and bacterial antigens (T-dependent, TI-1 and TI-2) can specifically influence the host mucosal immune response and resistance to potential nosocomial pathogens currently had been recently extended. It allows us to examine and characterize their protective and/or therapeutic properties against opportunistic agents. Monoassociated, genetically different mouse models are very useful for studying the role of various subsets of innate and adaptive immune cells in the generation of specific immune responses to bacterial antigens and pathogenic agents at the mucosal surface and should provide a new insight into the mechanisms of vaccine and probiotic protection and treatment of some infectious pathologies of human and/or animals.

Since it has become generally accepted that probiotics might be an alternative to an antibiotic method of prevention and treatment of opportunistic infections, we suggest that some innocuous gut colonizers will be appreciated for their roles in possibly ameliorating particular infections via stimulation of the natural GALT and NALT mucosal immune system, i.e., probiotic microbes have good prospects to be used as a complement to specific immunization.

We have been successful in studying host/bacteria local immune responses and the role of B-1 vs. B-2 cells in generating total IgA in NALT and GALT. Using formerly germfree (GF) C.B-17 SCID mice colonized by *Haemophillus influenzae* mutant strains with and without PC in their LPS structure as recipients for adoptively transferred, sorted B-1 or B-2 cells from the peritoneal cavity (PeC) of mice bearing different Ig allotypes (BALB/c of a-allotype and C.B-20 of b-allotype), we have shown that both B-1 cells and B-2 cells can develop IgA mucosal responses in draining lymph nodes (LNs) in NALT. Strikingly, IgA were specific to PC^+^ type 1 thymus-independent Ag (TI-1). Moreover, our data indicated that ‘bystander’ T cells are required for this local immune response. Only mice colonized with PC^+^ *H. influenzae* strain and reconstituted with a mixture of B-1 cells and non-B-1 PeC cells were able to develop a specific mucosal response to PC. The use of transferred donor cells with different Ig allotypes allowed us to examine contributions of B-1 vs. B-2 cells to local IgA production. Contribution of B-1 cells to anti-PC IgA response was significantly greater than of B-2 cells. Thus, activation of B-1 cells by PC was the first direct evidence of their selective stimulation by Ag via BCR.

We have also found that formerly GF BALB/c *imcomp* mice colonized with *Lactobacillus salivarius* developed both systemic and local [lamina propria (LP), mesenteric LN (MLN) and Peyer's patches (PP)] natural IgA responses. Interestingly, these bacteria showed an ability to translocate – disseminate into the internal parenchymal organs such as lungs, spleen, kidneys and liver after oral inoculation of GF IgA knockout (k.o.) mice, but not of their wild-type (W/T) B6 counterparts. However, colonization with Schaedler's *Escherichia coli* or *Morganella morganii* did not result in their translocation in either IgA k.o. or W/T mice and even protected against translocation of *L. salivarius* upon its subsequent oral inoculation. Increased level of autocrine growth factor Reg III β/γ expression was observed primarily in the caecum of colonized IgA deficient mice as compared to GF or *E. coli* monoassociated mice and gene expression of surfactant protein D (SP-D) had been induced in the lung of IgA deficient mice, inoculated by both bacteria and Schaedler's *E. coli* alone, and the number of macrophages and neutrophils were greatly increased in the bronchoalveolar lavage (BAL) of IgA-deficient mice colonized with *L. salivarius* on the third day after inoculation, as compared to GF mice or those monoassociated with *E. coli*. RegIII has been considered as an autocrine growth factor, but recently, this product found in secretory granules of Paneth cells has been shown to have in vitro bactericidal effects preferentially vs. gram-positive microbes.

The most conspicuous compartments of the cellular mucosal immune system of the gut include the intraepithelial (IEL) spaces, PP and the gut LP. Little is known about the role of gut microflora in the development and activation of elements of the cellular immune response compared to those of the humoral immune response. Generally, the approaches being used compare neonates and adults as well as GF and conventionally (CNV) reared adults, prior to and following their deliberate colonization with commensal microbes. We observed changes in total cell numbers and in the proportions of subsets of cells with different phenotypes and functions in the IEL compartment during normal development and after colonization of formerly GF adult mice with gut microbes. Monoassociation of GF SCID mice with Schaedler's *E. coli* led to an increase in the proportion of natural killer (NK) cells in the IEL spaces, compared to the cohort in Ag free (AF) *imcomp* mice, but below the level of those in CNV SCID mice. The functional ability and numbers of NK cells in the IEL compartment of GF SCID mice have not yet been reliably determined. It is likely that normal gut microbes can be partly responsible for the activated state of NK cells in the IEL compartment, compared to those in spleen and peripheral LNs. Further analysis is needed to investigate the mechanisms of humoral and cellular local and systemic responses of B-1 vs. B-2 cells to TI-1 and TI-2 Ags, such as PC (associated with polysaccharide carriers such as teichoic acids) and polyribitol phosphate (PRP) of encapsulated *H. influenzae*, type b. Anti-PRP IgM, but not IgA response was locally detectable in mice colonized with either PC^+^ or PC^−^ strains of *H*. *influenzae* after transfer of sorted and purified B-1 and CD4^+^ T cells into SCID recipients. In contrast, anti-PRP IgA was found in NALT fragment culture supernatants of recipients of unseparated PeC cells or mixtures of B-1 and non-B-1 PeC cells when mice were colonized with a PC^+^ strain of *H*. *influenzae*. Additional studies are required to determine the role of commensal bacteria in activation of APCs, T cells and NK cells as elements of protective immunity against opportunistic pathogens.

Since cytokine production is most informative evidence of regulating probiotic protective/therapeutic resistance to pathogens and bacterial translocation, we had investigated their possible roles in managing bacterially idiosyncratic/different stimulation of DCs. The level of anti-inflammatory IL-10 was induced by the treatment with *Bacillus subtilis* only; whereas the level of IL-12 had been stimulated by the treatment with Schaedler's *E. coli*, IL-1b was increased by the treatments of both LPS-containing strains: Schaedler's *E. coli* and *M. morganii*.

Selection of promising representatives of commensal bacteria, their antigenic structures and mutant strains with strong antibacterial, anti-inflammatory or immunomodulatory properties to be used for the construction of new bacterial preparations and/or targeted mucosal vaccines is important. In particular, it has been shown that the selected commensal bacteria exhibit a specific effect on opportunistic pathogens isolated in the clinical units. *L. salivarius*, Schaedler's *E. coli*, *M. morganii* and *B. subtilis* demonstrated both synergic stimulatory effect on host immune system functions and high inhibitory properties against *Klebsiella pneumoniae*, *Entrerobacter cloacae* and *Staphylococcus aureus*, but not to *Proteus mirabilis* or *Pseudomonas aeruginosa*.

# Sub article: Partially Purified Bacteriocin and Molecular Characterization Using 16S rRNA From Endemic Tropical Fruit Fermentation of Yellow Marquisa (*Passiflora edulis var. flavicarpa*) In Indonesia

**Published:** 2012-05-23

**Authors:** Sumaryati Syukur, Habibi Hidayat, Endang Purwati

**Affiliations:** 1 Laboratory of Biotechnology, Departement of Chemistry, Faculty of Math and Natural Sciences, University of Andalas, Padang, Indonesia, 25163; 2 Graduate Studies, Departement of Chemistry, Faculty of Math and Natural Sciences, University of Andalas, Padang, Indonesia, 25163; 3 Laboratory of Nutrition, Faculty of Animal Husbandary, University of Andalas padang, Indonesia, 25163

**Keywords:** Lactic acid bacteria, Bacteriocin, gene of 16S rRNA, Yellow Marquisa (*Passiflora edulis var. Flavicarpa*), *Weissella cibiria*

Yellow Marquisa (*Passiflora edulis var. flavicarpa)* is healthty tropical fruit with high nutrition contains, good taste and aromas. During spontaneous fermentation for 24 h, the pH was lower. Our investigation focus to isolate potential Lactic Acid bacteria (LAB), and partially purify anti microbial bacteriocin, and molecular species determination using 16 S rRNA. The medium of de Man, Ragosa, and Sharpe (MRS) were used to screen LAB, and 63 colonies were found. The screening of isolates based on LAB survival growth in acid pH ranges (2,3,4, and 5). Antimicrobial experiments were used using bacterial pathogen indicator such as *E. coli* NBRC 14237, *Staphylococcus aureus* NBRC 13276, *Bacillus subtilis* BTCCB, *Salmonela thypii*, *and Listeria monocytogenesis*), (Unv. Andalas Collection). Six isolate were conformed as antimicrobial pathogen and one isolate M4, selected as srong potential antimicrobial bacteriocin with diameter of inhibition zone reach (18 to 28 mm). Partially purified bacteriocin of potential M4 isolate, precipitated with 80% ammonium sulphate saturation and further purification by DEAE-Cellulose. The molecular weight determination by SDS-PAGES showed average of 10 kDa protein. The molecular determination were used primer of 27F: AGAGT TTGATCMTGGCTAG and R 1525: AAGGAGGTGWTCCARCC. The M4 isolate showing closely related with an homology of 98% with *Weissella cibiria* II-I-59 with accesion no NR 0369224,1, after complete sequence of PCR products (1427 bp). There is no report so far obtaining *Weissella cibiria* in yellow marquisa resistant low pH with highly antimicrobial bacteriocin.

# Sub article: The University Of Chicago Gnotobiotic Research Animal Facility (GRAF): a new member in the gnotobiotic community

**Published:** 2012-05-23

**Authors:** Betty R. Theriault, Christina Olivares, Alan Vest, George P. Langan

**Affiliations:** Animal Resources Center, The University Of Chicago, Chicago, IL, USA

## Introduction

Gnotobiology has a rich history of fluidity in providing technology for researchers to dissect complex biological questions. The University Of Chicago recently found itself on the crest of a new wave of research interests questioning existing paradigms in autoimmunity, allergy and gastrointestinal disorders. To assist researchers in their ability to dissect the multifactorial influences contributing to these disease pathways, we developed a Gnotobiotic Research Animal Facility (GRAF). The purpose of this abstract is to share The University Of Chicago's experience in developing a gnotobiotic research animal facility, lessons learned in establishing the facility and the future direction in research interests this technology is positioned to support.

## Approach

The University Of Chicago has extensive experience in using and maintaining semirigid isolators; however, in evaluating the request to maintain germ free mice, it soon became apparent that we would have to invest in flexible film isolators, the equipment to deliver autoclaved supplies to the isolators and learn the technology required to sustain mice in a germ free environment. We were generously provided training by a collaborator at another institution. After preparation, we received our first shipment of germfree Swiss Webster breeding pairs from a commercial source. Within 30 days of arrival, our first cohort of mice required euthanasia for what was later determined to be clinical signs resulting from hypovitaminosis. Recognizing we had maintained sterility of our first isolator, but had overlooked other measures of quality control and standard operating procedures, we set about gathering more knowledge and training before maintaining additional mice. Synergizing many of the principals of practice gained at three facilities visited, we started over. Our second shipment of germfree Swiss Webster mice was received, and in short order, our first litter of mouse pups were born (Fig. 1).

We continued to encounter success and expand our program from one isolator to three in the first year and seven operational isolators during the second. By the fourth year of operation, we had outgrown the initial space allocated to gnotobiotic/axenic isolators within our Gordon Center for Integrative Sciences (GCIS) and began preparation to construct a larger dedicated facility within our new Knapp Center for Biomedical Discovery (KCBD). Since initiating occupation of our new facility, we have encountered a 30% increase in isolator usage with projected increase of 100% during the next year (Fig. 2).

Although we have encountered three isolated bacterial contaminations over the course of our 4-year history, each contamination was traced to a source, and in each case, practices were modified in an effort to prevent repeat contaminations of the same nature. As advancing technologies become available in the fields of immunology, genetics, proteomics, metabolomics and human medicine, gnotobiotic technologies are expected to allow for novel and specific in vivo dissection of multifaceted components of complex diseases such as food allergy, immune-mediated diabetes, inflammatory bowel disease and celiac disease.

## Summary

The University Of Chicago has benefited from the tradition initiated by Dr. Philip Trexler in June 1960 by generously having gnotobiotic technology shared with us by institutions already established in this specialty field. The implementation of this technology required purchase of specialized equipment, training of specialized staff and implementation of programmatic practices. Although hurdles and setbacks were encountered, refinement and enhancements to the gnotobiotic program at The University Of Chicago have resulted in success and continued steady growth. Gnotobiotic technology is permitting several of our investigators to conduct research in areas of interest in human health and disease.

**Fig. 1:** Photographs detailing the initial phases of success in the maintenance of germfree mice at The University Of Chicago. (a) (top left) Example of the single level flexible film isolators in use at The University Of Chicago. (b) (bottom left) First Swiss Webster breeding colony maintained at The University Of Chicago. (c) (bottom right) First Swiss Webster litter born at The University Of Chicago.

**Fig. 2:** Gnotobiotic Research Animal Facility (GRAF) and GRAF growth chart with projections. (a) (upper left) GCIS component of GRAF with single level isolator tables only. (b) (upper right) KCBD component of GRAF. Isolator tables depicted in red are double level tables. Isolator tables in gray are also double level tables and not yet assigned. (c) (lower left) U of C Growth chart, which demonstrates history of germfree operations at the U of C, current space allocation and future projections.

# Sub article: Somatic cell nuclear transfer for genetic modification and preservation of gnotobiotic miniature pigs

**Published:** 2012-05-23

**Authors:** Hoon Taek Lee

**Affiliations:** Department of Animal Biotechnology, Bio-Organ Research Center/Animal Resources Research Center, Konkuk University, Seoul, Korea

Transgenically modified, gnotobiotic miniature pig is the most suitable organ donor for xenotransplantation in human. Introduction of complex genetic modification in these organisms can be best achieved by somatic cell nuclear transfer (SCNT). However, incomplete understanding of oocyte maturation mechanism, limited availability and high cost of receipting cytoplast are some of the barriers in the efficient application of SCNT to miniature pigs. Here, we show that recipient cytoplast from commercial Landrace pigs could successfully remodel and reprogram the donor nucleus from miniature pigs to form cloned embryos at a comparable efficiency. Transgenic embryos, expressing enhanced green fluorescent protein (EGFP) or LacZ, could also be produced from xenogenic nuclear transfer of cattle, mice and chicken cells into the enucleated oocytes of Landrace pigs, albeit at a lower frequency that depended on the phylogenic distance between the donor and recipient species. Introduction of transgene, however, did not affect the in vitro development competence of the cloned embryos. To further improve the efficiency of transgenic cloned animal production, we generated the stem-like cells from miniature pigs’ skin and testes. Testes-derived male germ-line stem cells (spermatogonial stem cells; SSC) could be maintained in culture for more than 2 months and could be reprogrammed to cloned embryos by SCNT. Although use of SSC did not improve the reprogramming efficiency and in vitro development of cloned embryos, it opens a new avenue for genetic manipulation of pigs. Furthermore, skin-derived stem-like cells may have application in tissue engineering. Pig embryos could also be successfully produced by SCNT of pig fibroblast or intracytoplasmic injection (ICSI) of boar sperm that were lyophilized and stored refrigerated at 4°C for long-term. The in vitro development of SCNT embryos could further be improved by inhibiting histone deacetylase in the cloned embryos for 24 h. Taken together, developments in SCNT technology may improve its utility for genetic modification and preservation of gnotobiotic miniature pigs to produce bio-organ for xenotransplantation in human.
